# Isomerization and Fragmentation Reactions on the [C_2_SH_4_] Potential Energy Surface: The Metastable Thione *S*-Methylide Isomer

**DOI:** 10.1021/acs.joc.0c02835

**Published:** 2021-01-27

**Authors:** Zoi Salta, Marc E. Segovia, Aline Katz, Nicola Tasinato, Vincenzo Barone, Oscar N. Ventura

**Affiliations:** † Scuola Normale Superiore, Piazza dei Cavalieri 7, 56126 Pisa, Italy; ‡ Computational Chemistry and Biology Group, CCBG, DETEMA, Facultad de Química, Universidad de la República, 11400 Montevideo, Uruguay

## Abstract

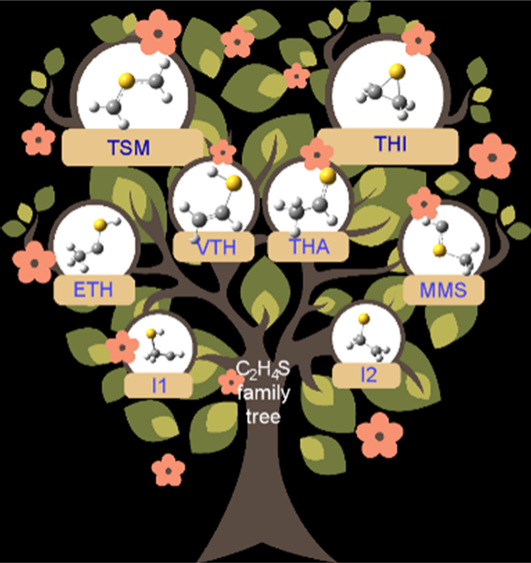

Thione *S*-methylide, parent species of the thiocarbonyl ylide family, is a 1,3-dipolar species on the [C_2_SH_4_] potential energy surface, not so much studied as its isomers, thiirane, vinyl thiol, and thioacetaldehyde. The conrotatory ring-closure reaction toward thiirane was studied in the 90s, but no complete analysis of the potential energy surface is available. In this paper, we report a computational study of the reaction scheme linking all species. We employed several computational methods (density functional theory, CCSD(T) composite schemes, and CASSCF/CASPT2 multireference procedures) to find the best description of thione *S*-methylide, its isomers, and transition states. The barrier from thiirane to thione *S*-methylide amounts to 52.2 kcal mol^–1^ (against 17.6 kcal mol^–1^ for the direct one), explaining why thiocarbonyl ylides cannot be prepared from thiiranes. Conversion of thiirane to vinyl thiol implies a large barrier, supporting why the reaction has been observed only at high temperatures. Fragmentations of thiirane to S(^3^P) or S(^1^D) and ethylene as well as decomposition to hydrogen sulfide plus acetylene were also explored. Triplet and singlet open-shell species were identified as intermediates in the fragmentations, with energies lower than the transition state between thiirane and vinyl thiol, explaining the preference of the latter at low temperatures.

## Introduction

Sulfur is a minor chemical constituent of our planet, with a concentration lower than 500 ppm in the Earth’s crust and much lower than one ppm in the atmosphere. However, its chemistry has a terrific impact on the biosphere in general and humankind in particular. Oxidation of naturally occurring sulfur compounds leads to sulfuric acid particles, which act as condensation nuclei for water, generating clouds and thus changing Earth’s albedo.^1^ Precipitation of this sulfuric acid droplets, in the form of acid rain, affects natural vegetation and crops. Sulfur, in the form of OCS, reaches the stratosphere to create the sulfate aerosol layer,^2^ affecting also Earth’s albedo. This layer is thought to provide even sites for heterogeneous reactions that could affect the ozone concentration. Direct injection of sulfur species by volcanoes in the stratosphere can also have significant meteorological impact. Those environmental issues have stimulated a large amount of research aimed to understand the atmospheric chemistry of sulfur and its compounds. The main topics investigated in several studies include the rates of emission, the chemical transformations, the transport of products and intermediate species from one site to another, and the removal of sulfur, mostly as sulfuric acid, back to the surface.^3^

In a different context, sulfur chemistry plays an important role in the Interstellar Medium (ISM). Nearly 20 sulfur-bearing compounds—roughly 10% of all the known compounds in this environment—were observed in the ISM and the circumstellar envelopes of evolved stars, ranging from diatomic SH^+^ and SH radicals^4,5^ to the C_2_H_5_SH species.^6^ The amount of sulfur-containing species in the ISM is consistent with the estimated cosmic abundance of sulfur^7^ and reproduced well by current astrophysical models.^8^ However, the same set of compounds accounts only for ∼0.1% of the expected sulfur abundance in the cold, dense clouds, and circumstellar regions around young stellar objects, with this fact remaining still unexplained.

**Scheme 1:**
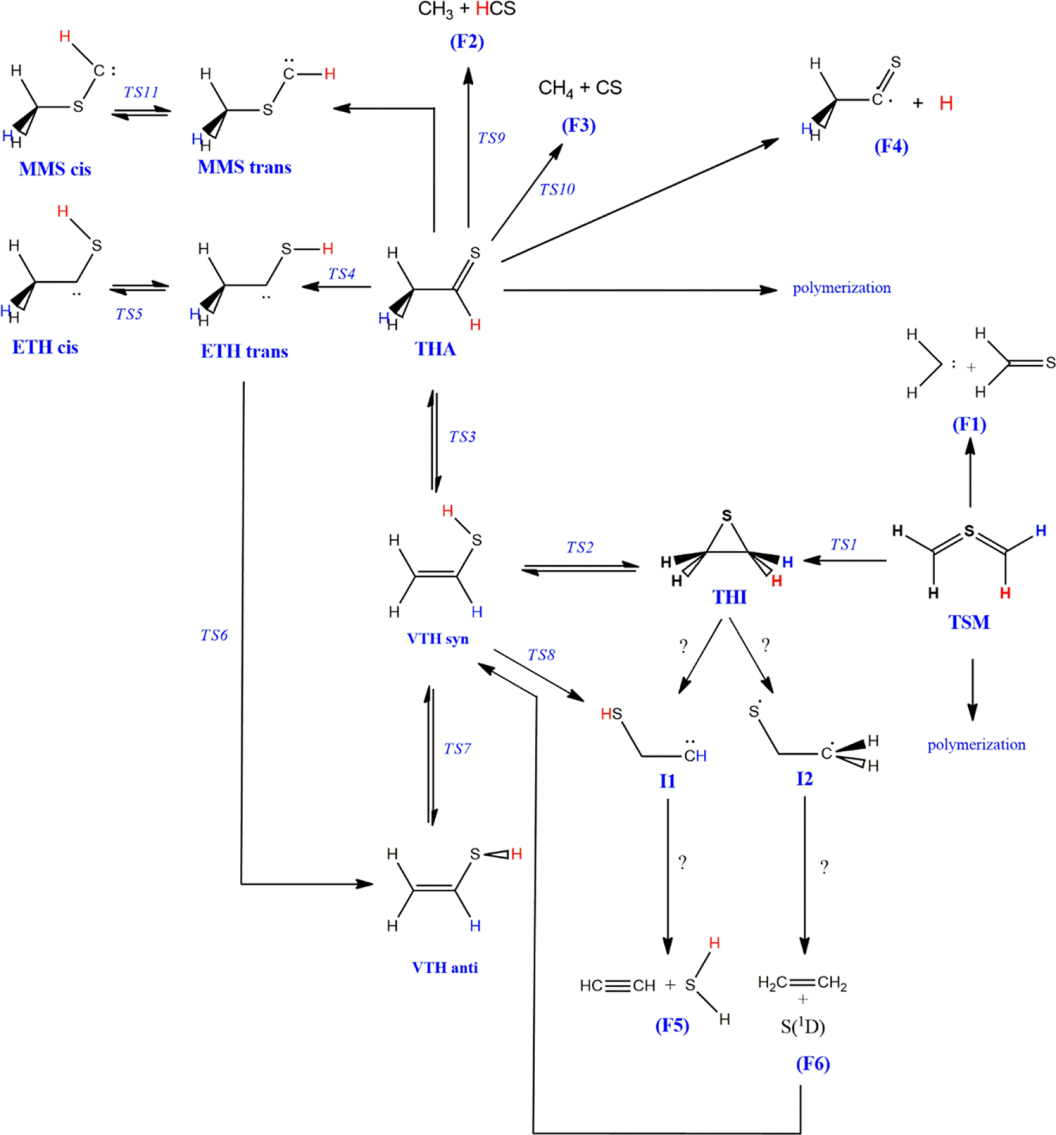
Relations between the Different Critical Points Found on the Potential Energy Surface

On these grounds, in the present paper, we analyze the potential energy surface (PES) for the family of compounds of chemical formula [C_2_H_4_S], which includes thioacetaldehyde (CH_3_CHS, THA), thiirane (*c*-C_2_H_4_S, THI), vinyl thiol (CH_2_CHSH, VTH), thione *S*-methylide (CH_2_SCH_2_, TSM), and carbenoid species like ethylidene thiol (CH_3_CSH, ETH) and methylidyne methyl sulfide (CH_3_SCH, MMS), together with their main fragmentation products (see Scheme 1). It is noteworthy that all the isovalent oxygen counterparts (acetaldehyde, oxirane, and vinyl alcohol for instance) are well characterized and have been actually detected in the ISM.^9−11^

Until its synthesis in 1965,^12^ vinyl thiol (VTH, the enolic form of thioacetaldehyde, THA) was unknown as a separate species and, in analogy with vinyl alcohol, its isovalent oxygen analog, was generally considered to be chemically unstable. It is now known that VTH is highly reactive at room temperature, but that, under suitable conditions, its lifetime can be extended to allow for spectral characterization.^13^ Being the simplest compound containing a SH group adjacent to a C=C double bond, VTH has been used as a model system for theoretical investigations on the π-donating ability of heteroatoms^14^ and on the importance of σ conjugative interaction in rotational isomerism.^15^ The conformation of this molecule, especially concerning internal rotation around the C–S bond, was studied experimentally and theoretically several times.^16−25^ Microwave and infrared spectroscopic data suggest that VTH exists in the planar *syn* (VTHs) and nearly planar *anti* (VTHa) conformations, with the *syn* conformer being the predominant species.^16−19^ Planar *trans* VTH is actually a transition state separating two equivalent VTHa conformers. VTH can be customarily produced by thermolysis of thiirane (THI) under reduced pressure in a flow system.^26^ One of the latest studies reports measurements of the pure rotational spectrum of the *syn* and *anti* isomers of VTH in their ground vibrational states up to 250 GHz. Using the new experimental data, Martin-Drumel et al.^27^ carried out searches on the spectral line survey EMoCA (Exploring Molecular Complexity with ALMA) performed toward the region of high-mass star formation Sagittarius (Sgr) B2(N). In the same work, the authors investigated also the energetics of the [C_2_H_4_S] isomers and their isovalent oxygen counterparts by high level quantum chemical calculations.

The three-member cyclic isomer THI itself was the subject of several experimental and theoretical studies,^13,28−32^ including thermal fragmentation and electrocyclic ring-opening. Two reaction channels were observed by Lown et al.^28^ for the former reaction. At temperatures below 250 °C, sulfur and ethylene are formed in stoichiometric yields, while rearrangement to VTH occurs at higher temperatures. The low-temperature reaction follows first-order kinetics, which was interpreted in terms of a pseudo-unimolecular mechanism involving a low-lying triplet excited state of THI.^28−30^ Thermal decomposition of THI was also investigated by Chin et al.^33^ in a flow system by following the changes of its photoelectron spectra at various temperatures. Since no observable changes were observed below 600 °C, the difference with the results of ref (28) was ascribed to the much smaller contact times in the flow system than in the static system used by Lown. At higher temperatures, conversion of THI into VTH and THA (not found before by Lown et al.^28^) was observed. Hydrogen sulfide, alkynes, carbon disulfide, and thiophene were detected as products of secondary reactions. Also, the decomposition toward H_2_S and acetylene was suggested, possibly through VTH as an intermediate.^34^

Sherwood et al.^13^ proposed a concerted pathway ruled by a bicyclic transition state to explain the reaction toward VTH. They did not consider an alternative stepwise pathway involving the homolytic breaking of the relatively weaker C–S bond as a first step. Furthermore, the other expected isomerization product, THA, was not detected in those studies, its absence being explained in terms of the quite close bond energies between the C–S single and double bonds^28^ or of the tendency of THA to polymerize.^35,36^

For those reasons, Chin et al.^33^ re-examined the thermal decomposition of THI by photoelectron spectroscopy, showing that, at temperatures above 600 °C, THA is produced together with VTH with the relative intensities of the ionization bands, suggesting production of the two isomers in comparable amounts. THA can be formed either by tautomerization of VTH, or by direct rearrangement of THI itself. A conventional kinetic analysis could not explain whether the reaction proceeds through a concerted pathway, i.e., by simultaneous bond making and breaking, or through a stepwise pathway involving radical species. Both ab initio and semiempirical MO methods were used to try to elucidate this conundrum, but the results were inconclusive because of the limited accuracy of the employed methods.^33^ The most recent study of THI involved velocity map imaging of the sulfur atoms by using photodissociation at 217 nm to reveal the internal state distribution of the co-product ethylene.^37^ Multireference calculations suggested that this photodissociation pathway is mediated by a hot, transient biradical (CH_2_CH_2_S), which strongly favors CH_2_ hindered rotations in the predissociation complex. This photochemical ring-opening mechanism was invoked to account for the vibrational features observed in the low-recoil region, which were attributed to the relaxation of triplet ethylene to the torsional saddle point on the ground state singlet surface.

Contrary to its oxygen analog, THA is an unstable species in the gas phase (half-life of about 10 s),^35^ which polymerizes readily, usually forming cyclic trimers. The monomeric species was isolated and characterized in 1974 by photoelectron spectroscopy,^35^ followed by several other spectroscopic studies,^38−42^ often combined with quantum chemical calculations.^43−46^ THA is a *C_s_* molecule with eclipsed methyl and carbonyl groups, whose thermal stability and unimolecular reaction pathways were studied by Ding et al.^47^

We reported previously a speculative path to TSM^48,49^ based on a detailed computational study of the H-abstraction path in the oxidation of dimethyl sulfide (DMS) by OH radicals under the hypothesis of high hydroxyl concentration. In that case, we postulated that a reaction channel could be open for the transformation of DMS into another isomer of this family, namely, thioformaldehyde *S*-methylide or thione *S*-methylide (TSM). This species was found to be an energy minimum, rearranging to the more stable THI isomer (37.5 kcal mol^–1^ below TSM) through a transition state lying at 13.7 kcal mol^–1^ above TSM (both values at the G4 level used in that paper).

The thiocarbonyl ylide structure, of which TSM is the parent species, was first recognized by Knott.^50^ A review of the synthetic effort to prepare thiocarbonyl ylides can be seen in the work by Kellog,^51^ and a study on the general reactivity of thiocarbonyl ylides was presented in a review by Huisgen et al.^52^ The parent thiocarbonyl methylide TSM was prepared by Hosomi et al. in 1986^53^ starting from chloromethyl trimethylsilylmethyl sulfide, although to the best of our knowledge, it has not yet been isolated. Mloston et al. in 1994, however,^54^ were able to separate the channels leading thermically to thiiranes from thiadiazolines for the reaction under photolysis in an organic glass matrix at 77 K or an Ar solid matrix at 10 K. In this case, they found a route to thiirane through the corresponding thiocarbonyl ylide, taking a strong UV maximum at λ ≈ 350 nm as distinctive of the 1,3-dipolar structure R_2_C=S^+^—CH_2_^–^.

Among the many C_2_SH_4_ isomers, only THI seems to be accessible thermally from TSM by means of an electrocyclic ring-closing reaction, the reverse of the ring-opening reaction studied by Snyder^55^ at the CNDO level and later by Fowler et al.^32^ and Fabian^56^ at the MO single- and multireference levels. Fowler et al.^32^ studied both conrotatory and disrotatory paths, concluding that the former one is preferred, with a reaction barrier of 51.3 kcal mol^–1^, from THI to TSM. They also predicted that THI is more stable than TSM by 34.7 kcal mol^–1^ including ZPE (39.1 kcal mol^–1^ without ZPE, in agreement with the value of 38.9 kcal mol^–1^ obtained by Fabian).^56^ Besides that ring-closing reaction, only the reaction-channel leading to thioformaldehyde and carbene appears open. Both reaction paths are reinvestigated in this work in order to establish the link to the other better studied isomers.

TSM might also undergo a bimolecular dimerization by a cycloaddition reaction, in a similar way to that of TSM and thioacetone *S*-methylide, studied by Sustmann et al.^57^ A comprehensive analysis of the dimerization process and its products will be addressed in a forthcoming publication.

## Methods

Density functional theory (DFT), as well as composite schemes based on the coupled-cluster ansatz with single and double excitations together with a perturbative treatment of triples, CCSD(T),^58,59^ were employed to study the structure and energetics of all the considered species.

DFT calculations were performed using the ωB97X-D,^60^ M06-2X,^61^ and B2PLYP^62,63^ density functionals, in conjunction with the cc-pVTZ^64^ and jun-cc-pV(T+*d*)Z^65^ basis sets. In order to account for dispersion interactions, M06-2X and B2PLYP were augmented by Grimme’s D3 semiempirical dispersion contribution,^66^ which has been applied with considerable success to a large amount of different systems, including dimers, large supramolecular complexes, and reaction energies/barriers as well as surface processes (see, for example, refs (66−68) and references therein). The CBS-QB3^69^ and G4^70^ composite methods were used in their original implementations, together with the more recent SVECV-F12^71^ and jun-ChS^72,73^ models. SVECV-F12 employs M062X-D3/cc-pVTZ geometries for CCSD(T)-F12 CBS calculations augmented by core-valence correlation corrections at the MP2/cc-pCVTZ level. On the other side, the jun-ChS approach employs B2PLYP-D3/jun-cc-pV(T+*d*)Z geometries for CCSD(T)/jun-cc-pV(T+*d*)Z energy computations augmented by MP2 evaluations of complete basis set and core-valence contributions. For some of the species, for which multireference calculations were deemed necessary, the CASSCF^74,75^ and CASPT2^76,77^ procedures were used, as described later in the text.

After geometry optimizations with very tight convergence criteria (e.g., 10^–4^ Å on Cartesian coordinates), the Hessians were inspected to assure the correct number of negative eigenvalues for each species. Analytical second derivatives were employed when available, whereas numerical derivatives of analytical gradients were used in the other cases. Intrinsic reaction coordinates (IRC)^78^ were used to ensure that each saddle point connects the correct reactants and products. All the calculations were performed using the Gaussian 16^79^ and Molpro 19^80^ programs.

## Results and Discussion

Scheme 1 shows the reaction paths we have considered in the present work for the isomerization and fragmentation reactions. Question marks are depicted instead of transition state labels in the cases where we were unable to find a proper transition state structure.

### About the Effect of the Chemical Model on the Geometry

Since all the composite methods used in this work employ geometries optimized at the DFT level, a first step in this study is to evaluate the accuracy of the different functionals used for geometry optimization. To this end, we have compared the geometries of the stable species present on the PES for which reliable semi-experimental structures are available, namely, ethylene (C_2_H_4_), acetylene (HCCH), carbon monosulfide (CS), thioformaldehyde (CH_2_S), methanethiol (CH_3_SH, MTH), hydrogen sulfide (H_2_S), thiirane (*c*-C_2_H_4_S, THI), and thioacetaldehyde (CH_3_CSH, THA). The results are collected in Table 1, while the reader is referred to refs (81−83) for a more detailed discussion on the accuracy of DFT methods in the prediction of structural (and spectroscopic) properties. Here, we limit ourselves to point out that additional polarization functions applied on second-row atoms, like in the jun-cc-pV(T+*d*) basis set, should be used in order to obtain improved performances with respect to geometries.

**Table 1:** Theoretical Equilibrium Structure (Å for Bond Lengths and deg for Bond Angles) for Selected Species on the TSM Potential Energy Surface and Comparison to Experimental Data

			*ae*-CCSD(T)^a^	ωB97X-D		M06-2X-D3		B2PLYP-D3	
species	param.	exp.	cc-pwCVQZ	cc-pVTZ	jun-cc-pV(T+*d*)Z	cc-pVTZ	jun-cc-pV(T+*d*)Z	cc-pVTZ	jun-cc-pV(T+*d*)Z
CS	r_CS_	1.535^b^	1.537	1.530	1.526	1.528	1.523	1.543	1.538
H_2_S	r_HS_	1.336^c^	1.335	1.341	1.338	1.339	1.336	1.340	1.337
	θ_HSH_	92.11^c^	92.3	92.7	92.7	92.4	92.3	92.5	92.5
C_2_H_2_	r_CC_	1.203^d^	1.204	1.194	1.194	1.194	1.194	1.202	1.203
	r_CH_	1.062^d^	1.062	1.063	1.063	1.063	1.063	1.061	1.061
C_2_H_4_	r_CC_	1.332^e^	1.331	1.323	1.322	1.322	1.322	1.327	1.328
	r_CH_	1.081^e^	1.081	1.083	1.083	1.082	1.082	1.081	1.081
	θ_HCC_	121.4^e^	121.5	121.6	121.6	121.6	121.6	121.6	121.6
CH_2_S	r_CS_	1.609^f^	1.610	1.603	1.599	1.601	1.597	1.614	1.610
	r_CH_	1.085^f^	1.085	1.087	1.084	1.087	1.087	1.086	1.086
	θ_HCS_	121.7^f^	121.9	122.0	122.0	122.0	122.0	122.0	122.0
CH_3_SH	r_CS_	1.818^g^	1.811	1.817	1.812	1.816	1.811	1.823	1.819
	r_CH_	1.104^g^	1.086	1.087	1.088	1.087	1.087	1.086	1.086
	r_SH_	1.329^g^	1.335	1.341	1.228	1.339	1.336	1.340	1.334
	θ_HCH_	110.3^g^	111.3	108.8	108.7	108.9	108.8	108.9	108.8
	θ_HSC_	100.3^g^	96.9	97.2	97.2	96.9	97.0	97.0	97.1
THI	r_CC_	1.481^f^	1.481	1.475	1.477	1.479	1.480	1.478	1.479
	r_CH_	1.080^f^	1.080	1.081	1.081	1.081	1.081	1.080	1.080
	r_CS_	1.811^f^	1.811	1.817	1.811	1.806	1.802	1.827	1.821
	θ_HCH_	115.7^f^	115.1	115.1	115.1	115.3	115.3	115.3	115.3
	θ_CSC_	48.25^f^	48.3	47.9	48.1	48.3	48.3	47.7	47.9
	θ_SCH_	114.98^f^	115.1	115.1	115.3	115.3	115.3	114.9	115.0
THA	r_CS_	1.610^h^	1.614	1.612	1.608	1.609	1.606	1.622	1.618
	r_CH_	1.089^h^	1.086	1.088	1.089	1.089	1.089	1.088	1.088
	r_CC_	1.506^h^	1.492	1.496	1.496	1.498	1.498	1.499	1.499
	θ_CCS_	125.3^h^	125.7	125.4	125.5	125.1	125.1	125.3	125.4
	θ_HCC_	119.4^h^	119.7	115.9	115.8	116.0	116.0	116.0	115.9
									
MARE^i^			0.40%	0.65%	0.90%	0.59%	0.62%	0.62%	0.54%
MAE len.^j^			0.003	0.006	0.011	0.005	0.006	0.006	0.004
MAE ang.^k^			0.69	1.14	1.15	1.07	1.06	1.10	1.00

a All electrons CCSD(T)/cc-pwCVQZ. This work from CS to CH_3_SH and ref (27) for THI and THA.

b Ref (88).

c Ref (89).

d Ref (90).

e Ref (81).

f Ref (91).

g Ref (92).

h Ref (93).

i Mean absolute relative error over all the structural parameters.^j^

j Mean absolute error over all bond lengths.

k Mean absolute error over all bond angles.

It is quite apparent that the ωB97X-D and M06-2X-D3 functionals tend to underestimate the C–S bond lengths whereas B2PLYP-D3 overestimates them. Although B2PLYP-D3 is generally more accurate, all methods are in fair agreement with the experimental structures, thus validating the use of both M06-2X-D3 and B2PLYP-D3 optimized geometries to calculate single-point energies using composite methods. Observe that the CBS-QB3 and G4 procedures include their own geometry optimization schemes, which rely on the B3LYP functional with 6-311G(2*d*,*d*,*p*) and 6-31G(2*df*,*p*) basis sets,^69^ respectively, sometimes introducing non-negligible errors in the final energies.^84,85^

THI and THA structures were recently optimized at a very high level of theory, CCSD(T,full)/cc-pwCVQZ, by Martin-Drumel et al.,^27^ in a work where also VTH was investigated. Therefore, their results are also included in Table 1 for comparative purposes (we performed calculations at the same level for the smaller species also). The accuracy of the different methods can be estimated from the overall mean absolute relative error (MARE) as well as from the mean absolute errors (MAE) for bond lengths and angles. The statistics reported in Table 1 show that, on average, the best performing functional is B2PLYP-D3 and that there is no real advantage in using a more sophisticated (and much more expensive) method for the geometry optimization. In fact, B2PLYP-D3 in conjunction with the jun-cc-pV(T+*d*)Z basis set shows a MARE slightly larger than the fully correlated CCSD(T)/pwCVQZ computations, with the MAE for bond lengths being very similar and that for bond angles only slightly worse (1.0° and 0.7° at B2PLYP-D3 and CCSD(T) levels, respectively). The optimized geometrical parameters computed for THI and THA at the CASPT2 level of theory are reported in Table S.1 of the Supporting Information, where they are compared against experimental data and also to DFT counterparts. The results obtained show that, for these two molecules, CASPT2 geometries are not more accurate than the DFT ones. Hence, the B2PLYP-D3 functional offers a remarkable cost/accuracy ratio for obtaining optimized geometries, on which more accurate energy calculations are performed. While the method performs very well for stable structures, a comprehensive benchmark of its performance for transition states is still lacking, though some recent studies report good results also in this connection.^86,87^

### The [C_2_H_4_S] Potential Energy Surface

**Figure 1:**
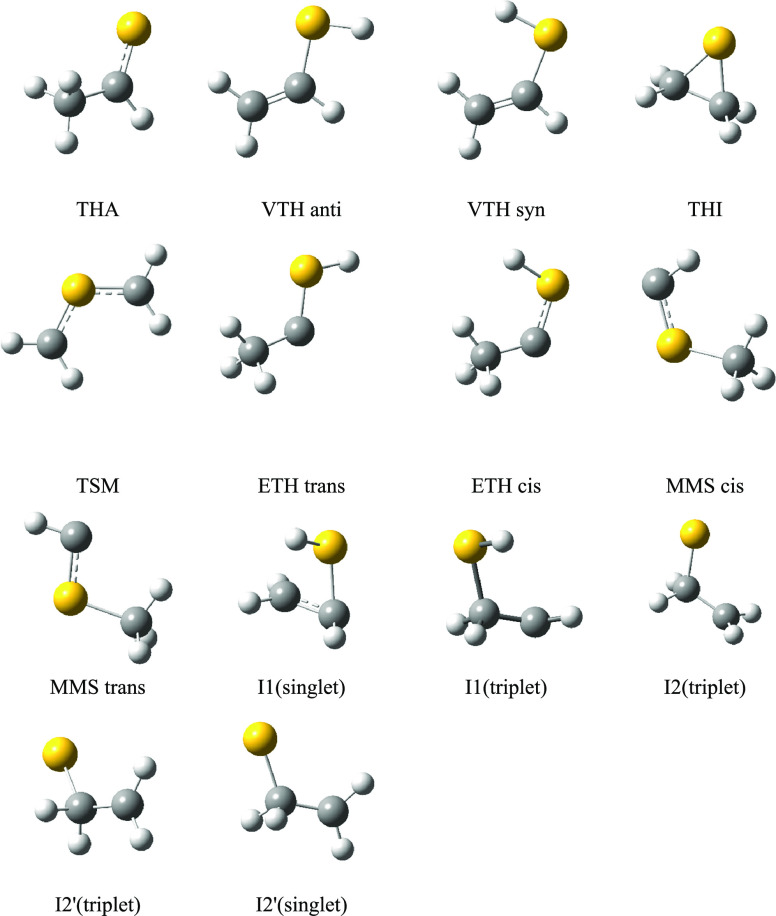
Structure of the unimolecular isomers on the [C_2_H_4_S] potential energy surface.

The stable species characterized on the [C_2_H_4_S] PES are shown in Figure 1, and their relative energies (with respect to that of TSM) are listed in Table 2. Cartesian coordinates for all the species are given in the Supporting Information, together with the absolute energy values.

**Table 2:** Relative Energies of the Species on the [C_2_H_4_S] PES with Respect to TSM, in kcal mol^–1^^c^

						**VTH**				**ETH**			
		**THI**		**THA**		**syn**		**anti**		**cis**		**trans**	
nethod	basis set	Δ(E+ZPE)	ΔH	Δ(E+ZPE)	ΔH	Δ(E+ZPE)	ΔH	Δ(E+ZPE)	ΔH	Δ(E+ZPE)	ΔH	Δ(E+ZPE)	ΔH
CBS-QB3		–35.1	–35.6	–35.7	–36.3	–35.2	–35.3	–35.1	–35.0	7.9	7.9	9.4	9.4
G4		–34.1	–34.7	–34.6	–35.2	–33.9	–34.0	–33.8	–33.7	8.5	8.6	10.1	10.1
M06-2X-D3	cc-pVTZ	–41.5	–42.0	–38.0	–38.5	–39.0	–39.0	–38.5	–38.5	5.3	5.4	6.5	6.7
	jun-cc-pV(T+*d*)Z	–38.8	–39.3	–35.4	–35.9	–36.4	–36.4	–36.0	–36.0	6.9	7.0	8.0	8.1
ωB97X-D	cc-pVTZ	–39.9	–40.4	–38.9	–39.3	–39.3	–39.4	–39.1	–39.0	3.8	5.9	5.3	5.4
	jun-cc-pV(T+*d*)Z	–36.9	–37.4	–36.0	–36.4	–36.5	–36.6	–36.4	–36.2	5.6	5.7	7.0	7.1
B2PLYP	cc-pVTZ	–35.5	–36.0	–36.5	–37.0	–36.3	–36.4	–36.0	–36.0	7.2	7.2	9.1	9.1
	jun-cc-pV(T+*d*)Z	–32.6	–33.1	–33.6	–34.2	–33.8	–33.9	–33.6	–33.5	8.7	8.8	10.5	10.6
SVECV-F12^a^	CBS	–35.1	–35.6	–35.0	–35.4	–36.3	–36.3	–34.7	–34.7	7.9	8.0	9.2	9.3
SVECV-F12^b^	CBS	–34.6	–35.2	–34.4	–35.0	–34.5	–34.7	–34.4	–34.3	8.4	8.4	9.9	9.9
Jun-ChS	CBS	–34.3	–34.8	–34.1	–34.6	–34.3	–34.4	–34.2	–34.1	8.8	8.8	10.3	10.3
CASPT2(9,4)	cc-pVTZ	–34.7		–35.1		–33.9		–31.9		14.3		12.1	

		**MMS**			
		**cis**		**trans**	
method	basis set	Δ(E+ZPE)	ΔH	Δ(E+ZPE)	ΔH
CBS-QB3		11.3	11.5	9.1	9.1
G4		11.6	11.8	9.4	9.4
M06-2X-D3	cc-pVTZ	9.6	9.8	7.4	7.5
	jun-cc-pV(T+*d*)Z	10.8	11.0	8.5	8.6
ωB97X-D	cc-pVTZ	8.7	8.9	6.8	6.9
	jun-cc-pV(T+*d*)Z	10.1	10.3	7.9	8.1
B2PLYP	cc-pVTZ	11.4	11.5	9.6	9.7
	jun-cc-pV(T+*d*)Z	12.6	12.8	10.7	10.7
SVECV-F12^a^	CBS	11.4	11.5	9.1	9.2
SVECV-F12^b^	CBS	11.9	12.0	9.7	9.7
Jun-ChS	CBS	12.0	12.2	9.8	9.8
CASPT2(9,4)	cc-pVTZ	16.8		11.9	

a Using M06-2X-D3/cc-pVTZ optimum geometries.

b Using B2PLYP/cc-pVTZ optimum geometries.

c Relative enthalpies at *T* = 298.15 K.

It is apparent that TSM is much less stable than any of the other classical isomers (THI, THA, VTH), but the relative stabilities of the latter depend on the specific model chemistry employed. G4 predicts the ordering THA < THI < VTH syn < VTH anti, while the SVECV-F12 and jun-Chs methods predict the ordering VTH *syn* < THI < THA < VTH *anti* if the M06-2X-D3/cc-pVTZ optimum geometries are used, and THI < VTH *syn* < VTH *anti* ≈ THA if the B2PLYP/cc-pVTZ geometries are used instead. Except for the case of VTH syn, the energy differences obtained using the two sets of geometries are small (less than 1 kcal mol^–1^), but the difference between isomers is even smaller, thus causing a reordering of the relative stabilities.

It is noteworthy that the multiconfigurational CASPT2/CASSCF(9,4) calculations predict the same ordering as G4. The energy difference between VTH *syn* and VTH *anti* obtained at the SVECV-F12//B2PLYP level is 0.1 kcal mol^–1^, whereas Martin-Drumel et al.,^27^ using a much more demanding HEAT-like scheme on top of CCSD(T,Full)/cc-pwCVQZ optimized geometries, obtained 0.12 kcal mol^–1^, and a value of 0.6 kcal mol^–1^ was reported by Almond et al.^23^ The stability order obtained in ref (27) is THA < THI ≈ VTH *syn* < VTH *anti*, with a range of about 1 kcal mol^–1^, quite close to our findings. The Jun-ChS and the SVECV-F12 results are very close with a stability order THI ≈ VTH *syn* < VTH *anti <* THA and a difference of 0.1 kcal mol^–1^ between VTH *syn* and VTH *anti.* The CASPT2 results are generally also close to them, except for VTH *anti*, where an unusual difference of more than 2 kcal mol^–1^ is observed. This might be due to a larger contribution of a multiconfigurational state, as discussed in a forthcoming section.

As shown in Scheme 1, two other carbenoid species have been located on the PES, produced respectively by an H-shift from carbon to sulfur (ETH), or a methyl shift between the same atoms (MMS). Both structures, which exhibit *cis-trans* isomerism, were obtained starting from the geometry of THA and are much less stable. The *cis* ETH structure is more stable than the *trans* conformer, both of them lying above TSM (by about 8.4 and 9.9 kcal mol^–1^ at the SVECV-F12//B2PLYP level). On the contrary, the MMS *trans* structure is more stable than the *cis*, again above TSM (by about 9.7 and 11.9 kcal mol^–1^ at the SVECV-F12//B2PLYP level). The stability of all these species is somewhat different (2–5 kcal mol^–1^) at the SVECV-F12 (or Jun-ChS) and CASPT2 levels, probably due to the presence of a low-lying doubly excited electronic configuration. We will discuss this point further when addressing the isomerization of THA in the next section.

### Unimolecular Isomerization and Fragmentation

As mentioned above, the only reasonable isomerization path for TSM is cyclization toward THI. The conrotatory ring-closure reaction of TSM toward THI was explored in a joint study with other species by Fabian,^56^ while the conrotatory vs disrotatory ring opening of THI has been previously studied by Fowler and Schaefer.^32^ As mentioned above, thermal reaction of THI was observed to branch at about 600 °C, below which fragmentation to sulfur plus ethylene was observed.^33^ At higher temperatures, however, THA and VTH were obtained as products.^26^ The lack of a ring-opening reaction to give TSM was attributed to the weakness of the C–S bond relative to the C–C one.

If TSM is described in terms of two double S=C bonds, then rotation around the C–S bond would exhibit multiconfigurational characteristics as discussed by Fowler et al.^32^ When the bond breaks upon rotation, a radical center is generated both on the carbon and sulfur atom, but the latter can also recover an electron from the other S–C “double” bond, reducing its oxidation state from 4 to 2 and generating an uncoupled electron on the second carbon. This would be an open-shell singlet state that requires a multideterminantal description. If TSM is instead described as a 1,2-dipole, like CH_2_=S(+)–CH_2_(−), then rotation around the single S–C bond should not be so demanding energetically and, at the same time, could be described by a single reference wavefunction.

DFT methods, being built in terms of the electronic density and not the wavefunction, could approximately cope with the situation independently of how large is the multireference character of TSM. The dipolar vs diradicaloid structures of TSM were discussed by Fabian^56^ with single and multireference CASPT2/CASSCF(4,4) procedures without reaching conclusive evidence and reporting that B3LYP provided essentially the same results concerning alternative reaction paths and reactivity parameters of pericyclic reactions. On the other side, Fowler et al.^32^ maintained that the inclusion of a two-configuration wavefunction to describe TSM was mandatory and at the highest theoretical level they employed, TZ2P TCSCF-CISD+Q, the THI-TMS energy difference was 39.1 kcal mol^–1^ (34.7 kcal mol^–1^ with the ZPE correction). Fabian^56^ obtained a value of 38.9 kcal mol^–1^ at the CASPT2 level (without ZPE). These values are in good agreement with those obtained at the SVECV-F12 level with the two different geometries, Jun-ChS and CASPT2 (35.1, 34.6, 34.3, and 34.7 kcal mol^–1^ including the ZPE). Therefore, it can be safely concluded that the TSM-THI energy difference is only marginally affected by non-dynamical correlation energy and not too large geometric distortions. Of course, the situation might be different for the transition state.

**Figure 2:**
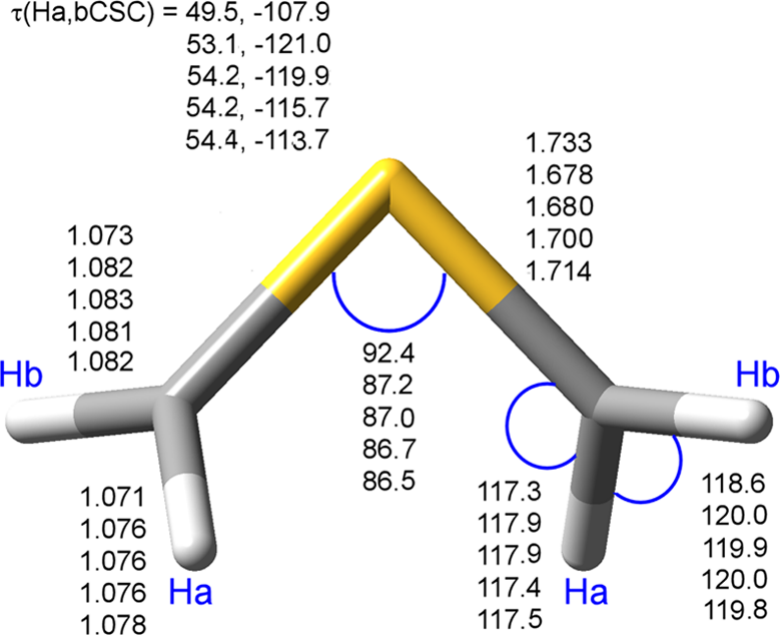
Geometries of the transition state TS1 between TSM and THI. From top to bottom, the entries for each parameter were calculated at the TCSCF TZ2P according to ref (31), and M06-2X-D3, ωB97X-D, B2PLYP-D3, and CASPT2/CASSCF(9,4) with the jun-cc-pV(T+*d*)Z basis set. Bond lengths are in Å, angles in degrees.

The structure of the TS1 transition state on the reaction path from TSM to THI is shown in Figure 2. We display there a comparison of the structure reported by Fowler et al.^32^ with those obtained by using the M06-2X-D3, ωB97X-D, and B2PLYP-D3 functionals, and the CASPT2/CASSCF(9,4) method, in all cases with the jun-cc-pV(T+*d*)Z basis set. In Table 3, we collect a summary of the energies and barriers for this transition state and reaction path as well as for the others in the isomerization processes, which will be discussed later.

**Table 3:** Relative Energies Δ(Ε+ZPE) with Respect to TSM of the Transition States for the Isomerization Reactions Showed in Scheme 1^e^

		**TS1**		**TS2**		**TS3**		**TS4**		**TS5**		**TS6**	
		**(TSM→THI)**		**(THI→VTHs)**		**(VTHs→THA)**		**(THA→ETHt)**		**(ETHt→ETHc)**		**(ETHt→VTHa)**	
method	basis set	Δ(E+ZPE)	barrier	Δ(E+ZPE)	barrier	Δ(E+ZPE)	barrier	Δ(E+ZPE)	barrier	Δ(E+ZPE)	barrier	Δ(E+ZPE)	barrier
CBS-QB3		16.0	16.0	19.1	54.2	20.6	55.7	37.5	73.2	38.8	29.4	29.2	19.8
G4		16.9	16.9	21.5	55.7	21.7	55.6	37.9	72.5	39.4	29.3	29.9	19.9
M06-2X-D3	cc-pVTZ	34.2	34.2	24.0	65.5	16.6	55.6	35.0	73.0	34.5	28.0	23.3	16.8
	jun-cc-pV(T+*d*)Z	21.6	21.6	26.9	65.7	19.3	55.7	25.2	60.6	36.6	28.7	25.2	17.2
ωB97X-D	cc-pVTZ	20.3	20.3	23.8	63.8	16.2	55.5	34.3	73.1	33.4	28.1	23.3	18.0
	jun-cc-pV(T+*d*)Z	21.5	21.5	27.0	64.0	19.1	55.7	36.6	72.6	35.9	28.9	25.5	18.5
B2PLYP	cc-pVTZ	18.5	18.5	24.7	60.2	18.4	54.7	36.2	72.7	38.0	29.0	27.6	18.5
	jun-cc-pV(T+*d*)Z	19.5	19.5	27.4	60.1	21.0	54.8	38.4	72.1	40.2	29.7	29.5	19.1
SVECV-F12^a^	CBS	16.9	16.9	28.0	63.0	20.6	54.9	37.0	72.0	38.1	28.8	28.5	19.3
SVECV-F12^b^	CBS	17.6	17.6	24.5	59.1	21.0	55.6	37.7	72.1	38.6	28.8	29.1	19.2
Jun-ChS	CBS	17.5	17.5	23.1	57.4	21.2	55.5	37.9	72.0	39.0	29.1	29.4	19.1
CASSCF/CASPT2	cc-pVTZ	15.7	15.7	28.9	63.6	20.8	55.9	37.3	72.4	30.4	65.4	30.4	18.2
CASSCF/CASPT2	6-31+G(d)^c^		15.0										
TCSF/CISD+Q	TZ2P^d^		13.2										

		**TS7**		**TS8**		**TS9**		**TS10**		**TS11**	
		**(VTHs→VTHa)**		**(VTHs→I1)**		**(THA→F2)**		**(THA→F3)**		**(MMSc→MMSt)**	
method	basis set	Δ(E+ZPE)	barrier	Δ(E+ZPE)	barrier	Δ(E+ZPE)	barrier	Δ(E+ZPE)	barrier	Δ(E+ZPE)	barrier
CBS-QB3		–33.1	2.1	41.5	76.7	57.5	93.3	45.0	80.7	51.3	39.9
G4		–31.7	2.2			58.3	92.9	46.4	81.0	52.1	40.4
M06-2X-D3	cc-pVTZ	–26.4	2.6	19.9	58.9	52.5	90.5	46.7	84.7	47.1	37.5
	jun-cc-pV(T+*d*)Z	–33.8	2.6	21.2	57.7	54.5	89.9	48.7	84.2	49.2	38.4
ωB97X-D	cc-pVTZ	–36.9	2.4	20.6	59.9	54.4	93.3	42.8	81.6	47.4	38.7
	jun-cc-pV(T+*d*)Z	–34.1	2.4	22.2	58.7	56.7	92.7	48.9	84.8	50.0	40.0
B2PLYP	cc-pVTZ	–33.7	2.6	22.0	58.2	57.3	93.8	44.1	80.6	51.5	40.1
	jun-cc-pV(T+*d*)Z	–31.2	2.6	25.1	58.9	59.3	93.1	46.3	80.1	53.6	41.1
SVECV-F12^a^	CBS	–32.8	2.5	24.1	60.4	57.7	92.7	46.9	81.9	50.9	39.6
SVECV-F12^b^	CBS	–32.3	2.3	25.4	59.9	58.2	92.6	46.6	81.0	51.4	39.5
Jun-ChS	CBS	–32.1	2.2	24.5	58.8	58.9	93.0	46.4	80.5	51.6	39.6
CASSCF/CASPT2	cc-pVTZ	–31.4	2.5	23.8	57.7	57.2	92.3	45.9	81.0	51.7	34.9

a Using M06-2X-D3/cc-pVTZ optimum geometries.

b Using B2PLYP/cc-pVTZ optimum geometries.

c Ref (56).

d Ref (32).

e Barriers to the reactant shown before the arrows are also given. Relative enthalpies at *T* = 298.15 K and all energies in kcal mol^–1^.

Our DFT calculations show two significant differences with respect to those at the TCSCF/TZ2P level. On the one side, Fowler’s structure is looser, with a longer S–C bond and a larger CSC angle. On the other side, the DFT calculations predict a smaller apical angle for the CH_2_ groups. These facts point toward a more (THI) product-like transition state in the case of Fowler’s calculations and a more (TSM) reactant-like transition state in the case of the DFT calculations. The M06-2X-D3 and ωB97X-D results are similar, while the B2PLYP results are slightly shifted toward the TCSCF/TZ2P ones. Fowler’s results do include the two-configuration wavefunction needed to describe correctly the rotation, but it does not include dynamical energy in the calculation. The DFT results, on the contrary, include a large fraction of such dynamical correlation but formally include only one determinant to represent the wavefunction. Nevertheless, this determinant is not built on top of independent particle functions, but the Kohn–Sham orbitals do already include correlation at least in principle. Therefore, the electronic density should incorporate the multiconfigurational character of the process. This implies that there are different reasons to argue in favor of the higher (or lower) accuracy of either set of calculations.

To resolve the discrepancies in the previous paragraph, we followed two lines of reasoning. On the one hand, we compared the more different geometrical parameters—the S–C bond length and the CSC angle—with the situation in both THI and TSM. In the first case, Fowler’s results (1.815 Å, 48.6°) have been compared with those here obtained at M06-2X-D3 (1.802 Å, 48.5°), ωB97X-D (1.811 Å, 48.1°), and B2PLYP-D3 (1.821 Å, 47.9°) functionals, all combined the jun-cc-pV(T+*d*)Z basis set. The already mentioned experimental results (1.815 Å and 48.3°) suggest that the TCSCF/TZ2P and ωB97X-D/jun-cc-pV(T+*d*)Z results are the most accurate. In the second case, Fowler’s parameters for TSM (1.647 Å and 113.7°) have to be compared with the same level of theory, meaning M06-2X-D3/jun-cc-pV(T+*d*)Z (1.615 Å, 115.5°), ωB97X-D/jun-cc-pV(T+*d*)Z (1.614 Å, 115.6°), and B2PLYP-D3/jun-cc-pV(T+*d*)Z (1.636 Å, 115.2°) results. It is quite apparent that, in analogy with the TS case, the B2PLYP results are closer to the TCSCF/TZ2P results than those of the two other functionals.

On the other hand, we performed a CASPT2/CASSCF geometry optimization of TSM, using the cc-pVTZ basis set. In the case of TSM (point group *C*_2*v*_), we included nine orbitals (3/4/1/1 for each symmetry) in the active space and correlated four electrons, for a total of 161 CSFs (configuration spin functions). The optimized geometry at this level shows a C–S bond length of 1.650 Å and an angle of 113.3°.

These results are in good agreement with those of Fowler et al.^32^ and may cast doubts about the use of DFT methods for the study of the reaction. Therefore, as a last attempt, we performed the geometry optimization of TS1 itself at the CASPT2/CASSCF level with the same active space and four correlated electrons. The results of this optimization are included as the last line for each parameter in Figure 2. It is observed that the structure issuing from inclusion of both dynamical and non-dynamical correlation is somehow tighter than the TCSCF/TP2Z counterpart, although not as tight as that predicted by M06-2X-D3 or ωB97X-D functionals. However, the B2PLYP-D3 results are very close to the CASPT2/CASSCF ones, thus suggesting that these geometries can be employed as a basis for the further calculation of more accurate energies. This is the same conclusion we reached in the analysis of the reference species in Table 1 and the structures of THI and TSM, which is also supported by recent results obtained for the reactivity of molecules containing the cyano group.^86,87,94^

Having then checked that both static and dynamic correlation do affect the geometry but, at the same time, that the B2PLYP-D3 method gives results close to the CASPT2 ones, we can proceed to analyze the energetics. We already saw that the SVECV-F12 method gives a very reasonable value for the TSM-THI energy difference. The TS1-TSM energy differences obtained with all the methods employed are shown in Table 3. Our most accurate values, G4 (16.9 kcal mol^–1^), SVECV-F12//M06-2X-D3 (16.9 kcal mol^–1^), SVECV-F12//B2PLYP (17.6 kcal mol^–1^), and jun-ChS (17.5 kcal mol^–1^) are considerably higher than those of Fowler et al.^32^ (13.0 kcal mol^–1^ at the TZP 2R CISD+Q level) and Fabian^56^ (15.0 kcal mol^–1^ at the CASSCF(4,4)/CASPT2 level with the 6-31G(d) basis set). Our own CASSCF(9,4)/CASPT2 calculations with the cc-pVTZ basis set gave a value of 15.7 kcal mol^–1^.

Thus, the values for the barrier show a quite significant spread (from 13.0 to 17.6 kcal mol^–1^ without considering DFT results). On the contrary, there is a good agreement among all these methods concerning the values of the barrier for the reverse process (THI to TSM). As detailed in the following, the origin of the problem can be traced back to the unbalanced description of static and dynamic correlation for TSM.

Comparing Fowler’s and Fabian’s results, we see that inclusion of both non-dynamical and dynamical correlation energy increases the barrier. A better description of the active space and an improvement of the basis set makes our CASPT2 value even a bit higher than Fabian’s at the same level, consistent with the previous observation. Finally, using the B2PLYP geometries, which are close to those issuing from multiconfigurational treatments, and the SVECV-F12 procedure, which includes higher levels of dynamical correlation energy than CASPT2, we got a slightly higher value of 17.6 kcal mol^–1^, which we consider the best estimate. In passing, it should be noted that the jun-ChS model delivers the same result as the SVECV-F12//B2PLYP method, without the need of resorting to explicitly correlated CCSD(T) computations. Hence, the jun-ChS method seems to offer the best compromise between accuracy and computational cost.

**Figure 3:**
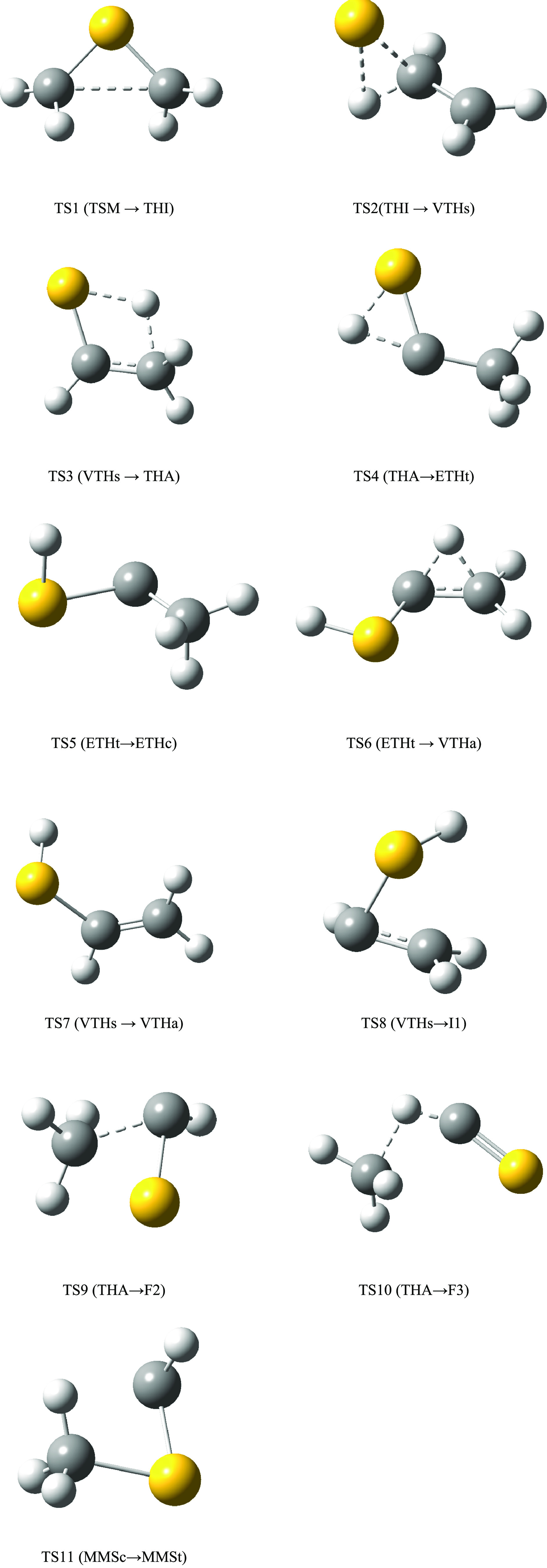
Structure of the transition states involved in the main isomerization processes.

The barrier for the transformation of TSM to THI (17.6 kcal mol^–1^ at the SVECV-F12//B2PLYP level) is relatively high but much lower than the reverse one (on account of the stability of THI with respect to TSM, the reverse barrier at the SVECV-12//B2PLYP level is 52.2 kcal mol^–1^; Fowler et al.^32^ reported 51.3 ± 4.0 kcal mol^–1^). Therefore, this explains why TSM and other thiocarbonyl ylides are not formed from the corresponding thiiranes. THI has only one other isomerization channel open, namely, the conversion to *syn* vinyl thiol (VTHs) through the transition state TS2 (see the relative energy and barrier in Table 3). Although another H-transfer is formally possible, all attempts to locate a transition state for the direct conversion THI → THA failed. As can be seen in Figure 3, TS2 is a monocylic TS (the distance of the sulfur atom from the tetrahedral carbon and the planar CH_2_ carbon are 1.858 and 2.724 Å, respectively), which could also represent the transition state for the direct insertion of a S(^1^D_2_) to ethylene, as proposed by Sherwood et al.^13^ (see later on).

**Figure 4:**
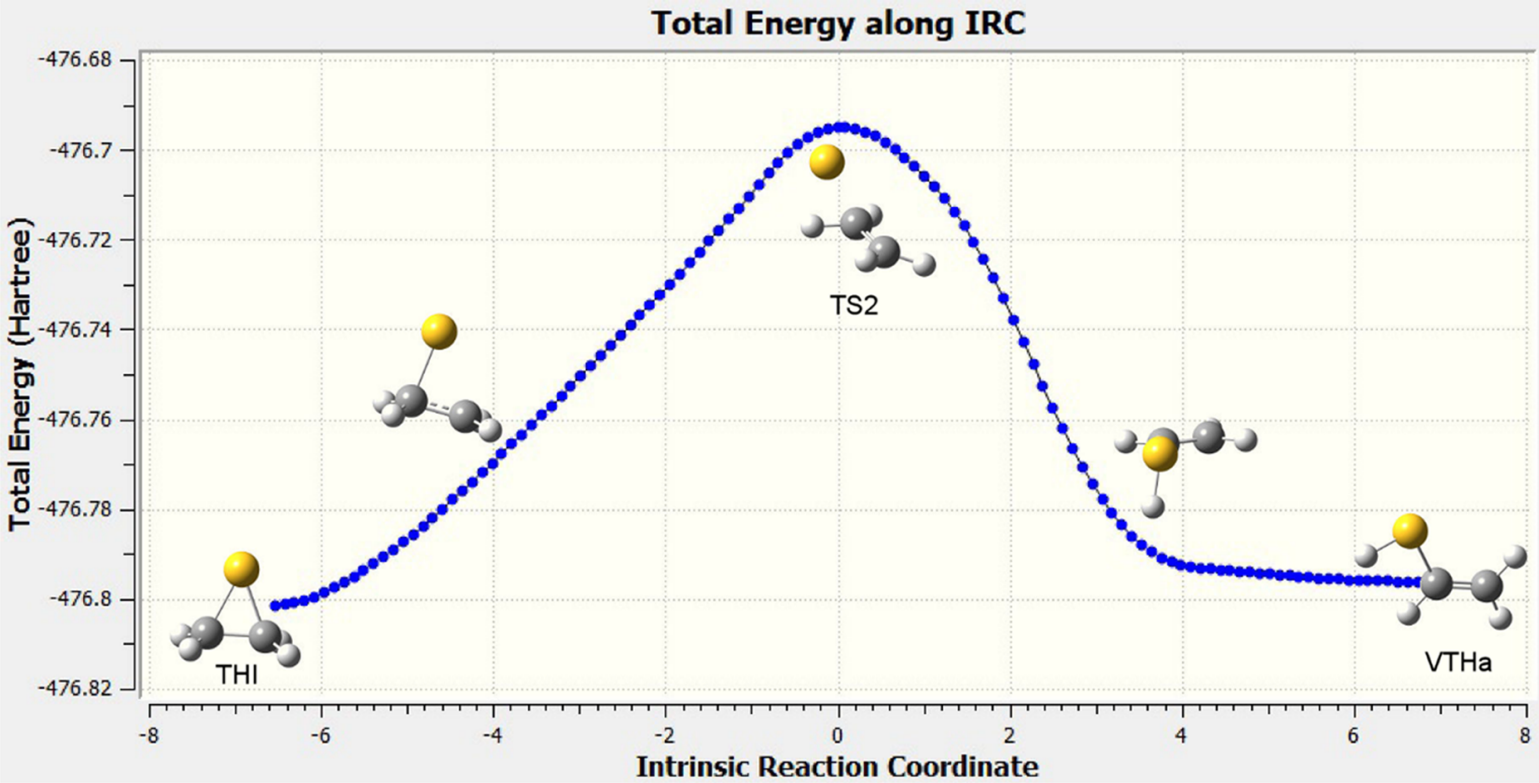
Total energy (in Hartree) along the intrinsic reaction coordinate (IRC), in steps of 0.05 Å in the forward and reverse directions (60 points each). Calculations at the ωB97X-D/cc-pVTZ level. Image created by using GaussView6 [ref (95)].

To distinguish which reaction path does actually TS2 belong to, we performed an IRC calculation, whose result can be seen in Figure 4. The IRC shows the initial breaking of one of the S–C bonds in the cyclic THI followed by a movement of the S toward one adjacent C–H bond and finally the insertion of S into this bond. Observe that, once the S atom is inserted, the structure is in the basin of the transition state TS7 (see Figure 3), which connects VTHs and VTHa. The final movement in the IRC is a rotation around the newly formed S–C bond to end up in the final VTHa product.

TS2 is actually at a high energy, 59.1 kcal mol^–1^ at the SVECV-F12//B2PLYP level, higher than that needed for the conversion of THI into TSM. In this last case, however, since the reverse reaction has a smaller barrier, the equilibrium would be displaced toward THI, unless TSM could undergo other reactions, for instance, dimerization. Another possibility is dissociation of TSM toward CH_2_ and CH_2_S, which we have called F1 in Scheme 1. Energetics of the fragmentation products are given in Table 4. The F1 fragments, where the methylene species is in the singlet state, lies 76.1 kcal mol^–1^ (at the SVECV-F12//B2PLYP level) over TSM and, since THI is 34.6 kcal mol^–1^ below it, the total energy required for such a decomposition is more than 110 kcal mol^–1^, making this path irrelevant since other reactions are open at lower energies. Curiously enough, S–CH_2_ reaction coordinate calculations, both with the M06-2X-D3 and the ωB97X-D methods, using the cc-pVTZ basis set, produced the CH_2_ fragment in a linear geometry, which formally corresponds to the open-shell singlet instead than the bent geometry of the closed shell carbene.

**Table 4:** Relative Energies Δ(Ε+ZPE) with Respect to TSM of the Species Involved in Fragmentation of THI and THA^e^

method	basis set	**F1**	**F2**	**F3**	**F4**	**F5**	**F6**	**THI triplet**	**I1 ^*d*^ singlet**	**I1 triplet**	**I2 triplet**	**I2′ singlet**	**I2′ triplet**
CBS-QB3		74.3	47.4	–6.3	53.4	–5.3	53.3	64.5	Nc	40.5			
G4		74.9	47.9	–5.9	54.3	–4.1	52.0	65.5	Nc	40.9	18.9		
M06-2X-D3	cc-pVTZ	74.9	46.5	–5.4	50.7	–7.9	56.6	59.7	19.8	31.9	11.4		
	jun-cc-pV(T+*d*)Z	76.8	48.4	–3.2	52.8	–5.5	60.3	61.0	20.9	34.4	14.4		
ωB97X-D	cc-pVTZ	74.7	44.3	–5.3	48.8	–7.0	57.9	58.0	20.8	32.2	9.8		
	jun-cc-pV(T+*d*)Z	77.0	46.5	–2.8	51.2	–4.3	62.0	59.7	22.3	35.0	13.2		
B2PLYP	cc-pVTZ	75.4	43.2	–7.4	50.0	–7.8	58.8	62.3	Nc	36.7	13.8		
	jun-cc-pV(T+*d*)Z	77.3	45.3	–5.0	52.2	–5.4	62.6	63.7	25.3	40.0	17.0		
SVECV-F12^a^	CBS	75.6	48.0	–5.0	56.4	–5.0	54.2	64.5	22.3	41.2	18.6		
SVECV-F12^b^	CBS	76.1	48.7	–4.5	54.5	–4.9	54.8	65.2	Nc	41.8	18.9		
Jun-ChS	CBS	76.5	49.4	–4.0	55.3	–4.6	55.5	62.3	24.6	41.1	19.3		
CASSCF/CASPT2^c^	cc-pVTZ								26.8	36.9	17.7	14.4	12.7

a Using M06-2X-D3/cc-pVTZ optimum geometries.

b Using B2PLYP/cc-pVTZ optimum geometries.

c The fragmentation products present no multireference character.

d “Nc” denotes no convergence.

e All energies in kcal mol^–1^.

As already mentioned, THI can undergo isomerization to VTH or fragmentation into a sulfur atom and ethylene (F6) or H_2_S and acetylene (F5). The fragmentations leading from THI to F5 and F6 are endothermic (by 29.7 and 89.6 kcal mol^–1^, respectively), whereas the isomerization to syn VTH is thermoneutral. However, kinetic aspects are more involved. Sherwood et al.^13^ explored the kinetics of the addition of S(^1^D_2_) to ethylene and found that both THI and VTH were formed by a pressure-dependent reaction. Previous studies^30,96,97^ concluded that addition occurs with a Δ*H* of 85.0 kcal mol^–1^, in reasonable agreement with our Δ(E+ZPE) of 89.6 kcal mol^–1^ (fragmentation F6 in Table 4). However, if this is true, then the energy needed for fragmentation is much larger than that needed to overcome TS2, in contrast with the experimental evidence that dissociation occurs at lower temperatures than isomerization. This implies the presence of some intermediates (labelled I1 and I2 in Scheme 1), lying below the energy of TS2.

Both singlet (^1^D) and triplet (^3^P) states of sulfur can react with ethylene. Asymptotically, the reaction with S(^3^P) leads to a lower fragmentation limit, while if the ground electronic state of THI has to be reached, then S(^1^D) should be the initial reactant. Experimental and theoretical studies have been performed on those reactions, and arguments about the participation of excited states of thiirane and/or fast intersystem crossing between the singlet and triplet species were presented.^13,31,33,37,97^ We discuss our data with respect to refs (37) and (97), which are the most recent ones and report the most sophisticated calculations. Leonori et al.^97^ used crossed-beam dynamic experiments, low-temperature kinetics experiments, and high-level ab initio calculations to investigate the reaction between S(^1^D) and ethylene. In the process, they presented PES for both singlet and triplet sulfur addition using CCSD(T)/cc-pV(T+*d*)Z//B3LYP/cc-pV(T+*d*)Z calculations. In the case of the singlet, THA is the most stable product followed by VTHt (1.7 kcal mol^–1^ above THA). The transition state separating THI and VTHt lies 58.0 kcal mol^–1^ over THI, in good agreement with the value of 59.1 kcal mol^–1^ issuing from our computations at the SVECV-F12//B2PLYP level (see Table 3). On the other side, fragmentation to S(^1^D) and ethylene was located at 82.9 kcal mol^–1^ over THI, well above the energy needed by isomerization to VTHt. Weeraratna et al.,^37^ on the other hand, used the XMS-CASPT2 method to calculate both the asymptotic limits and the potential energy curves of 15 states converging to different dissociation limits. An endothermic dissociation to S(^1^D) and ethylene was obtained (by 88.0 kcal mol^–1^), in very good agreement with our result of 89.2 kcal mol^–1^ (54.8 relative to TSM at the SVECV-F12//B2PLYP level, Table 4, plus 34.6 of TSM with respect to THI). The reaction of S(^3^P) with ethylene leads to the triplet state of THI, which can easily lead to the triplet open species CH_2_CH_2_S, with a very high transition state ruling its conversion to the most stable species, namely triplet THA. For the purpose of comparison, we calculated the lowest lying triplet state of THI (see Table 4) and found that it lies about 100 kcal mol^–1^ over the singlet state. The energy difference may seem surprising for a triplet-singlet gap but is supported by the calculations of Weeraratna et al.^37^ (see Fig. 2 in ref (37)).

The triplet of the open species CH_2_CH_2_S is simple to find computationally (I2 triplet, see Figure 1) but no singlet species with that geometry could be located in our calculations, even at the CASSCF/CASPT2 level, with all the attempts leading to THI. Thus, addition of S(^1^D) to ethylene does not seem to proceed through such an intermediate. One can see in Fig. 2 of Weeraratna et al.^37^ that the rich system of crossing potential energy curves (PECs) obtained when the sulfur atom approaches the ethylene center of mass perpendicularly to the CC bond. The crossing between the lowest ^1^A_1_ and ^3^B_1_ PECs occurs at about 2.6 Å, while a previous crossing between the ^3^B_1_ and ^3^B_2_ PECs occurs at about 1.7 Å. Although DFT calculations can in principle describe only the ground state PEC at a given distance, we tried to identify the crossings between the triplet and singlet curves calculated at these less sophisticated levels.

**Figure 5:**
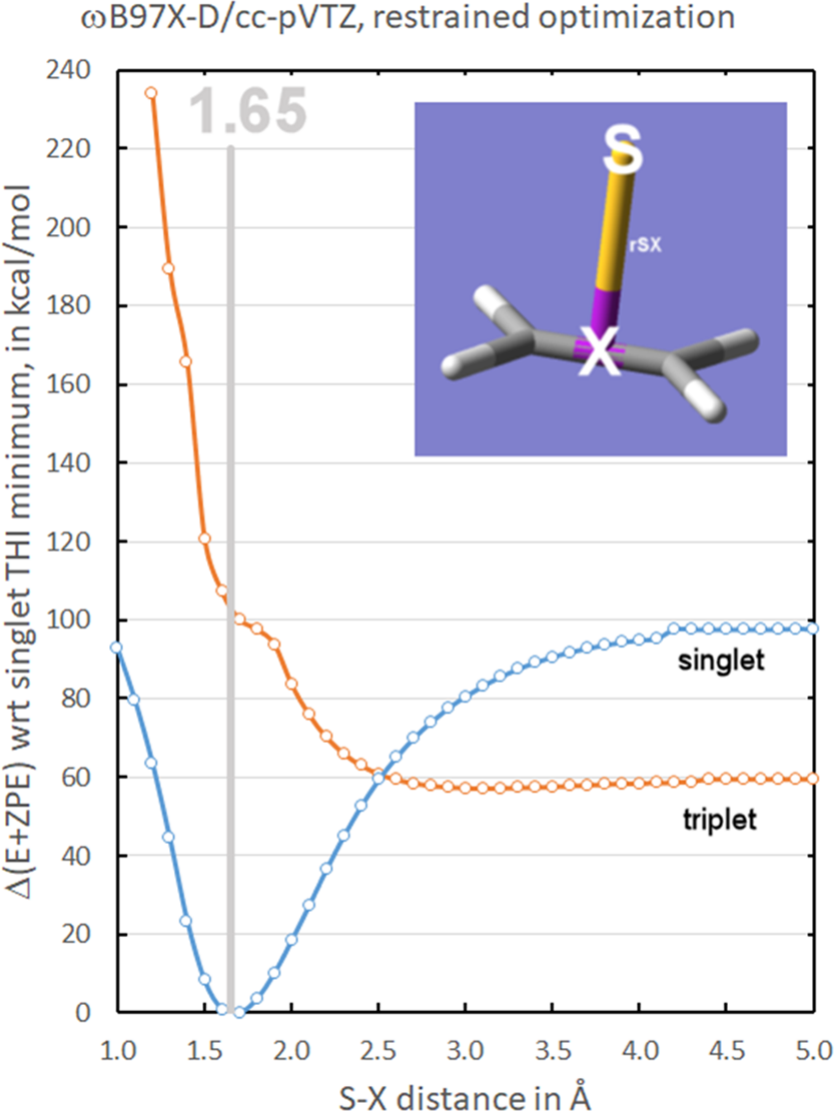
DFT calculation of the lowest singlet and triplet PECs, constraining the S atom to be perpendicular to the ethylene plane and using the S–X distance as the reaction coordinate.

The results sketched in Figure 5 show that crossing of the triplet and singlet curves occurs at 2.5 Å, while the shoulder indicating the crossing of the two triplet curves is found at 1.8 Å. Thus, it is clear that DFT methods can describe, at least semi-quantitatively, the features of the complicated singlet-triplet manifold of the fragmentation of THI. Weeraratna et al.^37^ suggested that the CH_2_CH_2_S biradical is the key transient structure linking the dissociation of THI to the more stable S(^3^P) + C_2_H_4_ asymptote, but we were unable to find such a species. The question is then if some different singlet and triplet states of the open structure can exist, different from the triplet I2 (^3^A″). One of the concepts utilized in those papers was that the collision between sulfur and ethylene could lead to excitation of the rotational modes of both methylene fragments. Following this idea, we were able to locate at the CASSCF/CASPT2 level two new states I2′ (a singlet and a triplet), which differ from I2 for the rotation of one of the CH_2_ fragments along the CC axis (see Figure 1). Both structures are very similar, the main difference being the position of the sulfur atom. Meanwhile, in the triplet state the S–C bond is 1.832 Å and the CCS angle is 113.0°, in the singlet state the bond distance is 1.784 Å and the angle is 121.5°. Observe that the geometries were fully optimized at the CASPT2/cc-pVTZ level, although they were not found at the DFT level, which converges to triplet I2 and singlet THI, respectively.

The final check of these structures is the energy difference (using the values of the CASSCF/cc-pVTZ method in Table 4) with respect to THI. The triplet is found at 47.4 kcal mol^–1^ above THI, whereas the singlet is located at 49.1 kcal mol^–1^, in both cases below the energy of TS2 at the same level of theory (63.6 kcal mol^–1^). This is a preliminary indication that a stationary point exists indeed on the PES lying below the energy of TS2, with this finding justifying the experimental observation that dissociation to sulfur and ethylene occurs at lower temperatures than isomerization to VTHs.

It has been shown experimentally that H_2_S and acetylene are major and minor products, respectively, of the thermal decomposition of thiirane.^33,34^ Ultraviolet photolysis of a mixture of H_2_S and acetylene is actually used for the preparation of THI,^18,19,23^ but it is unlikely that the fragmentation occurs directly without any intermediate. One reasonable hypothesis is that an intermediate biradical, similar to I2′, is formed upon the transfer of a hydrogen atom from one of the CH_2_ residues to the sulfur atom. If the resulting −SH group remains attached to the C atom from where the H was transferred, this route could lead to VTHs. If, on the contrary, the −SH remains attached to the −CH_2_ group, then a carbene −CH residue remains is obtained, in either an open singlet or a triplet state. We searched for this species using the triplet state as initial guess and found the structure I1(triplet) shown in Figure 1, which has the sought geometry, the cycle has been opened, and the distance from sulfur to carbon is large. In fact, a structure like that of I1(triplet) is expected upon transfer of an H atom from the −CH_2_ group in I2(triplet) to the sulfur atom. Starting from the triplet structure, we looked for the singlet, possibly in the excited closed-shell state, due to the single-reference methods we are using. Surprisingly, this was not the case, but the I1(singlet) structure turned out to be also a cycle (like THI) where sulfur is present as S(IV), sharing a double bond with the carbon atom in the −CH group. This species is actually 10.1 kcal mol^–1^ below the triplet, at the CASSCF(9,6)/CASPT2/cc-pVTZ level, and it is clearly a precursor for H_2_S + HCCH, after a second H transfer occurs. I1(singlet) is about 61.4 kcal mol^–1^ over THI at the CASSCF(9,6)/CASPT2/cc-pVTZ level, barely 2 kcal mol^–1^ below TS2. Although a more detailed study of the path from THI toward I1 to products is outside the scope of the present paper, the conclusion of this preliminary study is that there is some theoretical evidence to support the fragmentation of THI to S(^1^D) + ethylene at a lower energy (i.e., temperature) than that necessary for the isomerization to VTH, through a previously undescribed biradical intermediate. At the same time, it is possible that THI decomposes to H_2_S plus acetylene, without involving any open-shell species. I1 could also be reached starting from VTHsyn through the transition state TS8. This transition state is just about 1 kcal mol^–1^ above TS2, thus implying that I1, and therefore C_2_H_2_ + H_2_S, can be obtained from VTHsyn. The products, however, are thermochemically less stable than THI and would be produced, if at all, in much smaller amounts.

As discussed above, THI isomerizes to VTHs through the transition state TS2. VTHs and VTHa are connected through a low energy transition state TS7 (at 2.3 kcal mol^–1^ above VTHs) and the former can isomerize to THA through the transition state TS3, with a barrier of 55.6 kcal mol^–1^. We were unable to find a transition state connecting directly THI to THA, which is not unreasonable since such a TS would imply a hydrogen transfer between the carbon atoms, whereas the transition state between THI and VTH implies just a hydrogen transfer from carbon to sulfur, much easier to achieve. VTH and THA form part of a cycle, which includes also the ETH carbene. As can be seen in Scheme 1 and Figure 1, ETH has a carbenoid structure which, at variance with the other carbenoid structure I1(triplet) described above, has the −SH group attached to the carbenoid atom. As already mentioned, this structure, which presents a *cis*-*trans* isomerization, is even less stable than TSM (see Table 2). The transition state TS4 is located 72.1 kcal mol^–1^ over THA, thus implying that this reaction channel is probably closed and that THA would rather evolve toward VTHa since the barrier is actually much smaller (TS6 in Table 3).

In the same way as ETHt could be formed from THA if it overcomes transition state TS4 (TS1a in Ding et al.^47^), it could also undergo a methyl group migration from carbon to sulfur and end up giving MMS trans (MMSt). Surprisingly, the energy of this structure (both *cis* and *trans* conformers) is only slightly above the energy of the respective ETHc or ETHt conformers. However, we were unable to find a transition state for this interconversion. The barrier between THA and ETH (TS4) is 16.5 kcal mol^–1^ higher than the barrier for the conversion to VTH syn (TS3) and much lower than the energies necessary to overcome TS9 or TS10 for fragmentation. Thus, it is quite clear that THA will produce VTH, which is what is found experimentally.

Since all these isomerizations have large barriers, the reactions can occur only at high temperatures and it is possible then that fragmentation occurs. As can be seen in Table 4, all fragments are produced at much higher energies than TSM (and, therefore, THA) except F3, CH_4_ + CS, which is only 30 kcal mol^–1^ above THA. Note that CS has been found experimentally as one of the products in the pyrolysis of THI and/or THA and thus this reaction deserves further investigation. A transition state TS10 was found, but its barrier is about 81.0 kcal mol^–1^, implying that this channel is also less favorable than the isomerization to VTH, which seems to represent the only walkable route.

**Figure 6:**
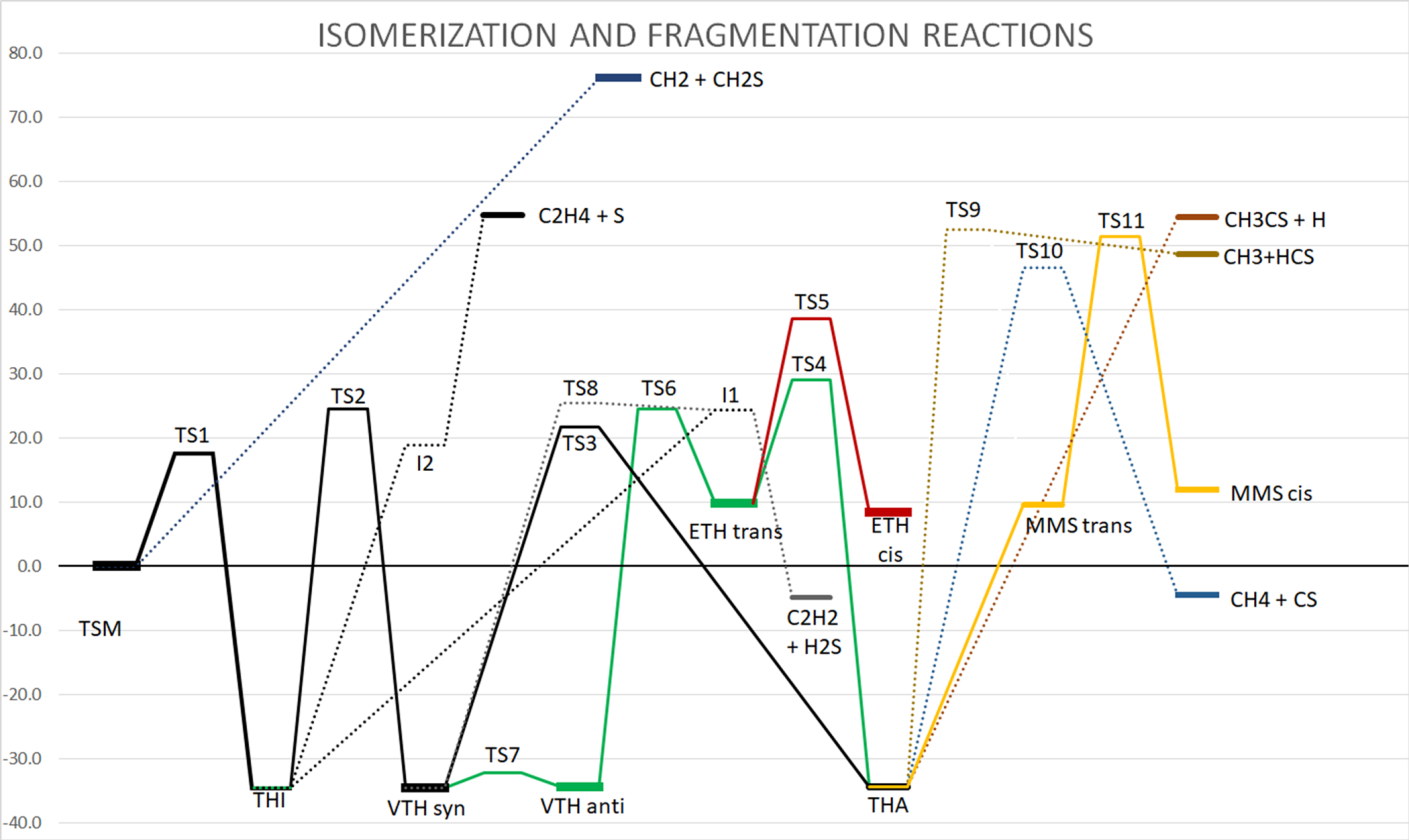
Schematic drawing of the relative energies (including ZPE) at the SVECV-F12//B2PLYP level, in kcal mol^–1^. Full lines represent the isomerization reactions, while dashed and dotted lines represent the fragmentations. The path in black is the most probable link between isomers.

All the previous results (which are graphically summarized in the PES shown in Figure 6) point toward a reasonably large stability of TSM, especially at low temperatures, with a barrier of about 15.7 kcal mol^–1^ toward THI, which can then either fragment or isomerize to VTH and THA.

## Conclusions

Density functional theory, CCSD(T)-based composite methods, and multireference MCSCF/CASPT2 calculations have been performed for all the critical points on the [C_2_H_4_S] potential energy surface. In particular, the relation between the elusive TSM isomer and the well-characterized THA, THI, and VTH isomers was analyzed for the first time together with the fragmentation of THA and THI and some intermediate structures with carbenoid or radicaloid characteristics.

A first result is that the barrier between TSM and THI is larger than previously assumed, 15.7 kcal mol^–1^ at the CASPT2/CASSCF level and 17.6 kcal mol^–1^ with the SVEC-F12//B2PLYP or Jun-ChS methods, to be compared with previous estimates ranging between 13.0 and 15.0 kcal mol^–1^. TSM is found to lie at 34.7 kcal mol^–1^ at the CASPT2/CASSCF level over THI, in close agreement with previous calculations, implying that the reverse barrier (from THI to TSM) is three times larger than the direct one, supporting the experimental finding that thiiranes can be obtained from thione *S*-methylides, but not the opposite.

THI itself is at the center of a complicated set of transformations. Although the first triplet state is less stable than the closed-shell ground state, intersystem crossing can occur at relatively large S–C distances. We found previously known and unknown intermediates that could lead either to sulfur plus ethylene or hydrogen sulfide plus acetylene products. However, the methods we employed, even the CASSCF/CASPT2 model, are not sufficiently accurate to determine unequivocally the relative order of these species and the transition states ruling their interconversion. On that basis, further studies are currently being pursued by our groups to elucidate better the role of the I1 intermediate in the decomposition of VTHsyn as well as the possibility of dimerization for TSM, which could introduce a completely different mechanism from the one concerning its transformation to THI.

## Supporting Information Available

XYZ coordinates of all species studied in this work (PDF)

## List of Acronyms

DFT: density functional theory
DMS: dimethyl sulfide
ETH: ethylidene thiol
I1: intermediate 1
I2: intermediate 2
I2′: intermediate 2′
IRC: intrinsic reaction coordinate
ISM: interstellar medium
MAE: mean absolute error
MARE: mean absolute relative error
MMS: methylidyne methyl sulfide
MTH: methanethiol
PEC: potential energy curve
PES: potential energy surface
THA: thioacetaldehyde
THI: thiirane
TS: transition State
TSM: thione *S*-methylide
VTH: vinyl thiol

## References

[ref1] Charlson R. J.; Lovelock J. E.; Andreae M. O.; Warren S. G. Oceanic Phytoplankton, Atmospheric Sulphur, Cloud Albedo and Climate. Nature 1987, 326, 655–661. 10.1038/326655a0.

[ref2] Crutzen P. J. The Possible Importance of CSO for the Sulfate Layer of the Stratosphere. Geophys. Res. Lett. 1976, 3, 73–76. 10.1029/GL003i002p00073.

[ref3] Tyndall R. S.; Ravishankara A. R. Atmospheric Oxidation of Reduced Sulfur Species. Int. J. Chem. Kinet. 1991, 23, 483–527. 10.1002/kin.550230604.

[ref4] Menten K. M.; Wyrowski F.; Belloche A.; Güsten R.; Dedes L.; Müller H. S. P. Submillimeter Absorption from SH^+^, a New Widespread Interstellar Radical, ^13^CH^+^ and HCl. Astron. Astrophys. 2011, 525, A77 10.1051/0004-6361/201014363.

[ref5] Neufeld D. A.; Falgarone E.; Gerin M.; Godard B.; Herbst E.; Pineau des Forêts G.; Vasyunin A. I.; Güsten R.; Wiesemeyer H.; Ricken O. Discovery of Interstellar Mercapto Radicals (SH) with the GREAT Instrument on SOFIA. Astron. Astrophys. 2012, 542, L6 10.1051/0004-6361/201218870.

[ref6] Kolesniková L.; Tercero B.; Cernicharo J.; Alonso J. L.; Daly A. M.; Gordon B. P.; Shipman S. T. Spectroscopic Characterization and Detection of Ethyl Mercaptan in Orion. Astrophys. J., Lett. 2014, 784, L7 10.1088/2041-8205/784/1/L7.

[ref7] Savage B. D.; Sembach K. R. Interstellar Abundances from Absorption-line Observations with the Hubble Space Telescope. Annu. Rev. Astron. Astrophys. 1996, 34, 279–329. 10.1146/annurev.astro.34.1.279.

[ref8] Neufeld D. A.; Godard B.; Gerin M.; Pineau des Forêts G.; Bernier C.; Falgarone E.; Graf U. U.; Güsten R.; Herbst E.; Lesaffre P.; Schilke P.; Sonnentrucker P.; Wiesemeyer H. Sulphur-bearing Molecules in Diffuse Molecular Clouds: New Results from SOFIA/GREAT and the IRAM 30 m Telescope. Astron. Astrophys. 2015, 577, A49 10.1051/0004-6361/201425391.

[ref9] Gottlieb C. A. Detection of Acetaldehyde in Sagittarius. Molecules in the Galactic Environment; Gordon M. A.; Snyder L. E. eds., John Wiley and Sons: 1973, 181.

[ref10] Dickens J. E.; Irvine W. M.; Ohishi O.; Ikeda M.; Ishikawa S.; Nummelin A.; Hjalmarson Å. Detection of Interstellar Ethylene Oxide (c-C_2_H_4_O). Astrophys. J. 1997, 489, 753–757. 10.1086/304821. 11541726

[ref11] Turner B. E.; Apponi A. J. Microwave Detection of Intestellar Vinyl Alcohol, CH_2_=CHOH. Astrophys. J. 2001, 561, L207–L210. 10.1086/324762.

[ref12] Strausz O. P.; Hikida T.; Gunning H. E. Photochemical Synthesis of Vinylthiols. Can. J. Chem. 1965, 43, 717–721. 10.1139/v65-095.

[ref13] Sherwood A. G.; Safarik I.; Verkoczy B.; Almadi G.; Wiebe H. A.; Strausz O. P. Unimolecular Isomerization of Chemically Activated Thiirane to Vinylthiol. J. Am. Chem. Soc. 1979, 101, 3000–3004. 10.1021/ja00505a029.

[ref14] Bernardi F.; Mangini A.; Epiotis N. D.; Larson J. R.; Shaik J. The π Donating Ability of Heteroatoms. J. Am. Chem. Soc. 1977, 99, 7465–7470. 10.1021/ja00465a012.

[ref15] Larson J. R.; Epiotis N. D.; Bernardi F. The Importance of *σ* Conjugative Interactions in Rotational Isomerism. J. Am. Chem. Soc. 1978, 100, 5713–5716. 10.1021/ja00486a021.

[ref16] Almond V.; Charles S. W.; MacDonald J. N.; Owen N. L. Ethenethiol: an Infared and Microwave Spectroscopic Study. J. Chem. Soc., Chem. Commun. 1977, 483–484. 10.1039/c39770000483.

[ref17] Tanimoto M.; Almond V.; Charles S. W.; MacDonald J. N.; Owen N. L. Microwave Spectrum and Conformation of Vinyl Mercaptan: The syn rotamer. J. Mol. Spectrosc. 1979, 78, 95–105. 10.1016/0022-2852(79)90037-7.

[ref18] Tanimoto M.; MacDonald J. N. Microwave Spectrum and Conformation of Vinyl Mercaptan: The anti rotamer. J. Mol. Spectrosc. 1979, 78, 106–119. 10.1016/0022-2852(79)90038-9.

[ref19] Almond V.; Charles S. W.; MacDonald J. N.; Owen N. L. Ethene Thiol: A Vibrational Analysis. J. Mol. Struct. 1983, 100, 223–239. 10.1016/0022-2860(83)90094-7.

[ref20] Samdal S.; Seip H. M. Potential for Rotation about C(*sp*^2^)–O and C(*sp*^2^)–S Bonds: Electron Diffraction Results for CH_2_ CH–OCH_3_ and CH_2_. J. Mol. Struct. 1975, 28, 193–203. 10.1016/0022-2860(75)80056-1.

[ref21] Kao J. Ab Initio Study of the Conformations of Vinyl Mercaptan, Methyl Vinyl Sulfide, and Methyl Allenyl Sulfide. J. Am. Chem. Soc. 1978, 100, 4685–4693. 10.1021/ja00483a011.

[ref22] Lister D. G.; Palmieri P. Ab Initio Study of the Rotational Isomlrism, Vibrational Force Field and Frequencies of Vinyl Mercaptan. J. Mol. Struct. 1978, 48, 133–138. 10.1016/0022-2860(78)87232-9.

[ref23] Almond V.; Permanand R. R.; MacDonald J. N. Internal Rotation and Framework Relaxation in Ethene Thiol. J. Mol. Struct. 1985, 128, 337–352. 10.1016/0022-2860(85)85009-2.

[ref24] Plant C.; MacDonald J. N.; Boggs J. E. Ab Initio Calculation of the Potential Functions for Internal Rotation Around the CS Bonds in Simple Ethene Thiols. J. Mol. Struct. 1985, 128, 353–363. 10.1016/0022-2860(85)85010-9.

[ref25] Plant C.; Boggs J. E.; MacDonald J. N.; Williams G. A. The Influence of Basis Set on the Ab Initio Prediction of Internal Rotation Barrier Heights in Ethene Thiol. Struct. Chem. 1992, 3, 3–8. 10.1007/BF00671974.

[ref26] Chin W. S.; Mok C. Y.; Huang H. H. Ethenethiol and 1-propene-1-thiol: a Photoelectron Spectroscopic Study. J. Electron Spectrosc. Relat. Phenom. 1994, 67, 173–179. 10.1016/0368-2048(93)02026-I.

[ref27] Martin-Drumel M.-A.; Lee K. L. K.; Belloche A.; Zingsheim O.; Thorwirth S.; Müller H. S. P.; Lewen F.; Garrod R. T.; Menten K. M.; McCarthy M. C.; Schlemmer S. Submillimeter Spectroscopy and Astronomical Searches of Vinyl Mercaptan, C_2_H_3_SH. Astron. Astrophys. 2019, 623, A167 10.1051/0004-6361/201935032.

[ref28] Lown E. M.; Sandhu H. S.; Gunning H. E.; Strausz O. P. Reactions of Sulfur Atoms. XI. Intermediacy of a Hybrid.pi.-thiacyclopropane in the Addition Reactions to Olefins and in the Thermal Decomposition of Episulfides. J. Am. Chem. Soc. 1968, 90, 7164–7165. 10.1021/ja01027a070.

[ref29] Strausz O. P.; Gosavi R. K.; Denes A. S.; Ciszmadia I. G. Molecular Orbital Calculations on the Ethylene Episulfide Molecule and its Isomers. Theor.Chim. Acta 1972, 26, 367–380. 10.1007/BF01036249.

[ref30] Strausz O. P.; Gunning H. E.; Denes A. S.; Ciszmadia I. G. The Reactions of Sulfur Atoms. XIV. *Ab Initio* Molecular Orbital Calculations on the Ethylene Episulfide Molecule and the S + C_2_H_4_ Reaction Path. J. Am. Chem. Soc. 1972, 94, 8317–8321. 10.1021/ja00779a006.

[ref31] McKee M. L. A Theoretical Study of Atomic Sulfur Reactions with Alkanes, Alkenes, and Alkynes. J. Am. Chem. Soc. 1986, 108, 5059–5064. 10.1021/ja00277a002.

[ref32] Fowler J. E.; Alberts I. L.; Schaefer H. F. III Mechanistic Study of the Electrocyclic Ring-Opening Reaction of Thiirane. J. Am. Chem. Soc. 1991, 113, 4768–4776. 10.1021/ja00013a009.

[ref33] Chin W. S.; Ek B. W.; Mok C. W.; Huang H. S. Thermal Decomposition of Thiirane and 2-Methylthiirane: An Experimental and Theoretical Study. J. Chem. Soc., Perkin Trans. 1994, 2, 883–889.

[ref34] Strausz O. P.; Gunning H. E.; Lown J. W. in Comprehensive Chemical Kinetics; C. H., Bamford; Tipper C. F. H. Eds., Vol. 5, Chap. 6, Elsevier: Amsterdam, 1972.

[ref35] Kroto H. W.; Landsberg B. M.; Suffolk R. J.; Vodden A. The Photoelectron and Microwave Spectra of the Unstable Species Thioacetaldehyde, CH_3_CHS, and Thioacetone, (CH_3_)_2_CS. Chem. Phys. Lett. 1974, 29, 265–269. 10.1016/0009-2614(74)85029-3.

[ref36] Karchmer J. H. The Analytical Chemistry of Sulfur and Its Compounds, Part 11; Wiley-Interscience: New York, 1972.

[ref37] Weeraratna C.; Amarasinghe C.; Joalland B.; Suits A. G. Ethylene Intersystem Crossing Caught in the Act by Photofragment Sulfur Atoms. J. Phys. Chem. A 2020, 124, 1712–1719. 10.1021/acs.jpca.9b11445. 31941276

[ref38] Judge R. H.; Moule D. C.; Bruno A. E.; Steer R. P. Thioketone Spectroscopy: An Analysis of the Lower Electronic Transitions in Thioacetone and Thioacetaldehyde. Chem. Phys. Lett. 1983, 102, 385–389. 10.1016/0009-2614(83)87061-4.

[ref39] Bruno A. E.; Moule D. C.; Steer R. P. Decay Dynamics of the Lowest Triplet and Lowest Excited Singlet States of Thioacetaldehyde and Thioacetone. J. Photochem. Photobiol., A 1989, 46, 169–180. 10.1016/1010-6030(89)80003-6.

[ref40] Judge R. H.; Moule D. C.; Bruno A. E.; Steer R. P. Thiocarbonyl Spectroscopy: Methyl Torsional Vibrations and Internal Rotational Barriers of Thioacetaldehyde in itsã^3^A‘ and X̃^1^ A’ states. J. Chem. Phys. 1987, 87, 60–67. 10.1063/1.453554.

[ref41] Moule D. C.; Bascal H. A.; Smeyers Y. G.; Clouthier D. J.; Karolczak J.; Niño A. An Analysis of the Methyl Rotation and Aldehyde Wagging Dynamics in the S 0 (X̃^1^ A’) and T 1 (ã^3^ A‘) states of Thioacetaldehyde from Pyrolysis Jet Spectra. J. Chem. Phys. 1992, 97, 3964–3972. 10.1063/1.462935.

[ref42] Smeyers Y. G.; Niño A.; Moule D. C. Dynamical and Spectroscopic Studies of Nonrigid Molecules. Application to the Visible Spectrum of Thioacetaldehyde. J. Chem. Phys. 1990, 93, 5786–5795. 10.1063/1.459574.

[ref43] Kroto H. W.; Landsberg B. M. The Microwave Spectrum, Substitution Structure, Internal Rotation Barrier, and Dipole Moment of Thioacetaldehyde, CH_3_CHS. J. Mol. Spectrosc. 1976, 62, 346–363. 10.1016/0022-2852(76)90275-7.

[ref44] Smeyers Y. G.; Niño A.; Bellido M. N. *Ab initio* Study of the Methyl Internal Rotation and Aldehyde Hydrogen Wagging of Thioacetaldehyde in the X^1^A’ and ã^3^A’’ States. Theor. Chim. Acta 1988, 74, 259–267. 10.1007/BF00530223.

[ref45] Weller T.; Klöpper D.; Köhler H.-J. Optimization of Molecular Geometries in the Pcilo and CNDO/2 Formalism. The barrier to Internal Rotation in Thioacetaldehyde. 1975, 36, 475–477, 10.1016/0009-2614(75)80283-1.

[ref46] Bachrach S. M.; Salzner U. Topological Electron Density Analysis of Organosulfur Compounds. J. Mol. Struct. 1995, 337, 201–207. 10.1016/0166-1280(94)04117-B.

[ref47] Ding W.-J.; Fang W.-H.; Liu R.-Z. Theoretical Studies on Unimolecular Reactions of Thioacetaldehyde. J. Mol. Struct.: THEOCHEM 2004, 682, 29–35. 10.1016/j.theochem.2004.05.022.

[ref48] Salta Z.; Lupi J.; Barone V.; Ventura O. N. H-Abstraction from Dimethyl Sulfide in the Presence of an Excess of Hydroxyl Radicals. A Quantum Chemical Evaluation of Thermochemical and Kinetic Parameters Unveils an Alternative Pathway to Dimethyl Sulfoxide. ACS Earth Space Chem. 2020, 4, 403–419. 10.1021/acsearthspacechem.9b00306.

[ref49] Salta Z.; Lupi J.; Tasinato N.; Barone V.; Ventura O. N. Unraveling the Role of Additional OH-Radicals in the H–Abstraction from Dimethyl Sulfide Using Quantum Chemical Computations. Chem. Phys. Lett. 2020, 739, 136963 10.1016/j.cplett.2019.136963.

[ref50] Knott E. B. Compounds Containing Sulphur Chromophores. Part I. The Action of Bases on Heterocyclic Sulphide Quarternary Salts. J. Chem. Soc. 1955, 916–927. 10.1039/jr9550000916.

[ref51] Kellogg R. M. The Molecules R_2_CXCR_2_ Including Azomethine, Carbonyl and Thiocarbonyl Ylides. Their Syntheses, Properties and Reactions. Tetrahedron 1976, 32, 2165–2184. 10.1016/0040-4020(76)85131-9.

[ref52] Huisgen R.; Fulka C.; Kalwinsch I.; Xingya L.; Mloston G.; Moran J. R.; Pröbstl A. Recent Developments of the Chemistry of Thiocarbonyl Ylides. Bull. Soc. Chim. Belg. 1984, 93, 511–532. 10.1002/bscb.19840930701.

[ref53] Hosomi A.; Matsuyama Y.; Sakurai H. Chloromethyl Trimethylsilylmethyl Sulphide as a Parent Thiocarbonyl Ylide Synthon. A Simple Synthesis of Dihydro- and Tetrahydro-thiophenes. J. Chem. Soc., Chem. Commun. 1986, 1073–1074. 10.1039/c39860001073.

[ref54] Mloston G.; Romanski J.; Schmidt C.; Reisenauer H. P.; Maier G. Photochemical and Thermal Generation of Thiocarbonyl Ylides from 2,5-Dihydro-1,3,4-thiadiazoles. Chem. Ber. 1994, 127, 2527–2530. 10.1002/cber.19941271226.

[ref55] Snyder J. P. Organo-sulfur mechanisms. III. Oxathiiranes. Differential Orbital Correlation Effects in the Electrocyclic Formation of Sulfur-containing Three-membered Rings. J. Am. Chem. Soc. 1974, 96, 5005–5007. 10.1021/ja00822a059.

[ref56] Fabian J. A theoretical Study of Some Non-classical Organic Compounds With Di-coordinated Sulfur. J. Mol. Struct.: THEOCHEM 1997, 398-399, 411–416. 10.1016/S0166-1280(96)04968-8.

[ref57] Sustmann R.; Sicking W.; Huisgen R. Thioformaldehyde S-Methylide and Thioacetone S-Methylide: An Ab Initio MO Study of Structure and Cycloaddition Reactivity. Chem. – Eur. J. 2003, 9, 2245–2255. 10.1002/chem.200204658. 12772299

[ref58] Purvis G. D. III; Bartlett R. J. A Full Coupled-Cluster Singles and Doubles Model: The Inclusion of Disconnected Triples. J. Chem. Phys. 1982, 76, 1910–1918. 10.1063/1.443164.

[ref59] Raghavachari K.; Trucks G. W.; Pople J. A.; Head-Gordon M. A Fifth-order Perturbation Comparison of Electron Correlation Theories. Chem. Phys. Lett. 1989, 157, 479–483. 10.1016/S0009-2614(89)87395-6.

[ref60] Chai J.-D.; Head-Gordon M. Long-range Corrected Hybrid Density Functionals with Damped Atom–atom Dispersion Corrections. Phys. Chem. Chem. Phys. 2008, 10, 6615–6620. 10.1039/b810189b. 18989472

[ref61] Zhao Y.; Truhlar D. G. The M06 of Density Functionals for Main Group Thermochemistry, Thermochemical Kinetics, Noncovalent Interactions, Excited States, and Transition Elements: Two New Functionals and Systematic Testing of Four M06-class Functionals and 12 Other Functionals. Theor. Chem. Acc. 2008, 120, 215–241. 10.1007/s00214-007-0310-x.

[ref62] Grimme S. Semiempirical Hybrid Density Functional with Perturbative Second-order Correlation. J. Chem. Phys. 2006, 124, 034108 10.1063/1.2148954. 16438568

[ref63] Biczysko M.; Panek P.; Scalmani G.; Bloino J.; Barone V. Harmonic and Anharmonic Vibrational Frequency Calculations with the Double-Hybrid B2PLYP Method: Analytic Second Derivatives and Benchmark Studies. J. Chem. Theory Comput. 2010, 6, 2115–2125. 10.1021/ct100212p. 26615939

[ref64] Kendall R. A.; Dunning T. H. Jr.; Harrison R. J. Electron Affinities of the First-row Atoms Revisited. Systematic Basis Sets and Wave Functions. J. Chem. Phys. 1992, 96, 6796–6806. 10.1063/1.462569.

[ref65] Papajak E.; Zheng J.; Xu X.; Leverentz H. R.; Truhlar D. G. Perspectives on Basis Sets Beautiful: Seasonal Plantings of Diffuse Basis Functions. J. Chem. Theory Comput. 2011, 7, 3027–3034. 10.1021/ct200106a. 26598144

[ref66] Grimme S.; Antony J.; Ehrlich S.; Krieg H. A Consistent and Accurate Ab Initio Parametrization of Density Functional Dispersion Correction (DFT-D) for the 94 Elements H-Pu. J. Chem. Phys. 2010, 132, 154104 10.1063/1.3382344. 20423165

[ref67] Tasinato N.; Grimme S. Unveiling the Non-covalent Interactions of Molecular Homodimers by Dispersion-corrected DFT Calculations and Collision-induced Broadening of Ro-vibrational Transitions: Application to (CH_2_F_2_)_2_ and (SO_2_)_2_. Phys. Chem. Chem. Phys. 2015, 17, 5659–5669. 10.1039/C4CP05680A. 25623466

[ref68] Sure R.; Grimme S. Comprehensive Benchmark of Association (Free) Energies of Realistic Host–Guest Complexes. J. Chem. Theory Comput. 2015, 11, 3785–3801. 10.1021/acs.jctc.5b00296. 26574460

[ref69] Montgomery J. A. Jr.; Frisch M. J.; Ochterski J. W.; Petersson G. A. A Complete Basis Set Model Chemistry. VI. Use of Density Functional Geometries and Frequencies. J. Chem. Phys. 1999, 110, 2822–2827. 10.1063/1.477924.

[ref70] Curtiss L. A.; Redfern P. C.; Raghavachari K. Gaussian-4 Theory. J. Chem. Phys. 2007, 126, 084108 10.1063/1.2436888. 17343441

[ref71] Ventura O. N. SVECV-F12: A Composite Scheme for an Accurate and Cost Effective Evaluation of Reaction Barriers. I. Benchmarking Using the HTBH38/08 and NHTBH38/08 Barrier Heights Databases Unpublished, preprint available at ChemRxiv.org; https://chemrxiv.org/articles/SVECVF12_A_Composite_Scheme_for_an_Accurate_and_Cost_Effective_Evaluation_of_Reaction_Barriers_IBenchmarking_Using_the_HTBH38_08_and_NHTBH38_08_Barrier_Heights_Databases/11770023

[ref72] Puzzarini C.; Barone V. Extending the Molecular Size in Accurate Quantum Chemical Calculations: The Equilibrium Structure and Spectroscopic Properties of Uracil. Phys. Chem. Chem. Phys. 2011, 13, 7189–7197. 10.1039/c0cp02636k. 21409277

[ref73] Alessandrini S.; Barone V.; Puzzarini C. Extension of the “Cheap” Composite Approach to Noncovalent Interactions: The jun-ChS Scheme. J. Chem. Theory Comput. 2020, 16, 988–1006. 10.1021/acs.jctc.9b01037. 31860293

[ref74] Werner H.-J.; Knowles P. J. A Second Order Multiconfiguration SCF Procedure with Optimum Convergence. J. Chem. Phys. 1985, 82, 5053 10.1063/1.448627.

[ref75] Kreplin D. A.; Knowles P. J.; Werner H.-J. Second-order MCSCF Optimization Revisited I. Improved Algorithms for Fast and Robust Second-order CASSCF Convergence. J. Chem. Phys. 2019, 150, 194106 10.1063/1.5094644. 31117783

[ref76] Werner H.-J. Third-order Multireference Perturbation Theory The CASPT3 Method. Mol. Phys. 1996, 89, 645–661. 10.1080/002689796173967.

[ref77] Finley J.; Malmqvist P.-Å.; Roos B. O.; Serrano-Andrés L. The Multi-state CASPT2 Method. Chem. Phys. Lett. 1998, 288, 299–306. 10.1016/S0009-2614(98)00252-8.

[ref78] Fukui K. The Path of Chemical Reactions - The IRC Approach. Acc. Chem. Res. 1981, 14, 363–368. 10.1021/ar00072a001.

[ref79] Frisch M. J.; Trucks G. W.; Schlegel H. B.; Scuseria G. E.; Robb M. A.; Cheeseman J. R.; Scalmani G.; Barone V.; Petersson G. A.; Nakatsuji H.; Li X.; Caricato M.; Marenich A. V.; Bloino J.; Janesko B. G.; Gomperts R.; Mennucci B.; Hratchian H. P.; Ortiz J. V.; Izmaylov A. F.; Sonnenberg J. L.; Williams Young D.; Ding F.; Lipparini F.; Egidi F.; Goings J.; Peng B.; Petrone A.; Henderson T.; Ranasinghe D.; Zakrzewski V. G.; Gao J.; Rega N.; Zheng G.; Liang W.; Hada M.; Ehara M.; Toyota K.; Fukuda R.; Hasegawa J.; Ishida M.; Nakajima T.; Honda Y.; Kitao O.; Nakai H.; Vreven T.; Throssell K.; Montgomery J. A. Jr.; Peralta J. E.; Ogliaro F.; Bearpark M. J.; Heyd J. J.; Brothers E. N.; Kudin K. N.; Staroverov V. N.; Keith T. A.; Kobayashi R.; Normand J.; Raghavachari K.; Rendell A. P.; Burant J. C.; Iyengar S. S.; Tomasi J.; Cossi M.; Millam J. M.; Klene M.; Adamo C.; Cammi R.; Ochterski J. W.; Martin R. L.; Morokuma K.; Farkas O.; Foresman J. B.; Fox D. J. Gaussian 16; Revision C.01.; Gaussian Inc.: Wallingford CT., 2016.

[ref80] Werner H.-J.; Knowles P. J.; Knizia G.; Manby F. R.; Schütz M.; Celani P.; Györffy W.; Kats D.; Korona T.; Lindh R.; Mitrushenkov A.; Rauhut G.; Shamasundar K. R.; Adler T. B.; Amos R. D.; Bennie S. J.; Bernhardsson A.; Berning A.; Cooper D. L.; Deegan M. J. O.; Dobbyn A. J.; Eckert F.; Goll E.; Hampel C.; Hesselmann A.; Hetzer G.; Hrenar T.; Jansen G.; Köppl C.; Lee S. J. R.; Liu Y.; Lloyd A. W.; Ma Q.; Mata R. A.; May A. J.; McNicholas S. J.; Meyer W.; Miller T. F. III; Mura M. E.; Nicklass A.; O’Neill D. P.; Palmieri P.; Peng D.; Pflüger K.; Pitzer R.; Reiher M.; Shiozaki T.; Stoll H.; Stone A. J.; Tarroni R.; Thorsteinsson T.; Wang M.; Welborn M. MOLPRO; version 2019.2, a package of ab initio programs see https://www.molpro.net.

[ref81] Penocchio E.; Piccardo M.; Barone V. Semiexperimental Equilibrium Structures for Buinding Blocks of Organic and Biological Molecules : The B2PLYP Route. J. Chem. Theory Comput. 2015, 11, 4689–4707. 10.1021/acs.jctc.5b00622. 26574259

[ref82] Barone V.; Ceselin G.; Fusé M.; Tasinato N. Accuracy Meets Interpretability for Computational Spectroscopy by Means of Hybrid and Double-Hybrid Functionals. Front. Chem. 2020, 8, 584203 10.3389/fchem.2020.584203. 33195078 PMC7645164

[ref83] Boussessi R.; Ceselin G.; Tasinato N.; Barone V. DFT Meets the Segmented Polarization Consistent Basis Sets: Performances in the Computation of Molecular Structures, Rotational and Vibrational Spectroscopic Properties. J. Mol. Struct. 2020, 1208, 127886 10.1016/j.molstruc.2020.127886.

[ref84] Curtiss L. A.; Redfern P. C.; Raghavachari K. Assessment of Gaussian-4 Theory for Energy Barriers. Chem. Phys. Lett. 2010, 499, 168–172. 10.1016/j.cplett.2010.09.012.

[ref85] Spada L.; Tasinato N.; Bosi G.; Vazart F.; Barone V.; Puzzarini C. On the Competition Between Weak O–H···F and C–H···F Hydrogen Bonds, in Cooperation with C–H···O Contacts, in the Difluoromethane – tert-butyl Alcohol Cluster. J. Mol. Spectrosc. 2017, 337, 90–95. 10.1016/j.jms.2017.04.001. 28919646 PMC5597040

[ref86] Salta Z.; Tasinato N.; Lupi J.; Boussessi R.; Balbi A.; Puzzarini C.; Barone V. Exploring the Maze of C_2_N_2_H_5_ Radicals and Their Fragments in the Interstellar Medium with the Help of Quantum-Chemical Computations. ACS Earth Space Chem. 2020, 4, 774–782. 10.1021/acsearthspacechem.0c00062.

[ref87] Puzzarini C.; Salta Z.; Tasinato N.; Lupi J.; Cavallotti C.; Barone V. A Twist on the Reaction of the CN Radical with Methylamine in the Interstellar Medium: New Hints from a State-of-the-art Quantum-chemical Study. Mon. Not. R. Astron. Soc. 2020, 496, 4298–4310. 10.1093/mnras/staa1652.

[ref88] Huber K. P.; Herzberg G. Molecular Spectra and Molecular Structure. IV. Constants of Diatomic Molecules; Van Nostrand Reinhold, 1979.

[ref89] Cook R. L.; De Lucia F. C.; Helminger P. Molecular Force Field and Structure of Hydrogen sulfide. Recent Microwave results. J. Mol. Struct. 1975, 28, 237–246. 10.1016/0022-2860(75)80094-9.

[ref90] Tamassia F.; Cané E.; Fusina L.; Di Lonardo G. The Experimental Equilibrium Structure of Acetylene. Phys. Chem. Chem. Phys. 2016, 18, 1937–1944. 10.1039/C5CP05997F. 26687993

[ref91] Penocchio E.; Mendolicchio M.; Tasinato N.; Barone V. Structural Features of the Carbon-sulfur Chemical Bond: A Semi-experimental Perspective. Can. J. Chem. 2016, 94, 1065–1076. 10.1139/cjc-2016-0282. 28912608 PMC5595238

[ref92] Herzberg G. Electronic Spectrum and Electronic Structure of Polyatomic Molecules; Van Nostrsand: New York, USA, 1966.

[ref93] Kutchitsu K. Structure of Free Polyatomic Molecules. Basic Data; Springer: Berlin, Germany, 1990.

[ref94] Baiano C.; Lupi J.; Tasinato N.; Puzzarini C.; Barone V. The Role of State-of-the-art Quantum-chemical Calculations in Astrochemistry: Formation Route and Spectroscopy of Ethanimine as a Paradigmatic Case. Molecules 2020, 25, 2873 10.3390/molecules25122873. PMC7357107 32580443

[ref95] Dennington E. D. II; Keith T. A.; Millam J. M. GaussView 6.0.16; Semichem, Inc. 2016

[ref96] Sidhu K. S.; Lown E. M.; Strausz O. P.; Gunning H. E. The Reactions of Sulfur Atoms. VI. The Addition to C_4_ Olefins. A Stereospecific Triplet-State Reaction. J. Am. Chem. 1966, 88, 254–263. 10.1021/ja00954a014.

[ref97] Leonori F.; Petrucci R.; Balucani N.; Casavecchia P.; Rosi M.; Skouteris D.; Berteloite C.; Le Picard S. D.; Canosa A.; Sims I. R. Crossed-Beam Dynamics, Low-Temperature Kinetics, and Theoretical Studies of the Reaction S(^1^D) + C_2_H_4_. J. Phys. Chem. A 2009, 113, 15328–15345. 10.1021/jp906299v. 19761231

