# Supplementary material for: Isomerization and Fragmentation Reactions on the [C2SH4] Potential Energy Surface: The Metastable Thione S-Methylide Isomer
Source: J Org Chem. 2021 Jan 27;86(3):2941–56. doi: 10.1021/acs.joc.0c02835 (PMC8023414; doi:10.1021/acs.joc.0c02835)
Supplement: Supplementary file 1 — jo0c02835_si_001.pdf [file jo0c02835_si_001.pdf]

Isomerization and fragmentation reactions on the [C_2SH_4] Potential Energy Surface. The metastable thione-S-methylide isomer.

Zoi Salta[1*], Marc E. Segovia[2], Aline Katz[2], Nicola Tasinato[1], Vincenzo Barone[1] and Oscar N. Ventura[2]
[1]*Scuola Normale Superiore, piazza dei Cavalieri 7, 56126 Pisa, Italy*
[2]*Computational Chemistry and Biology Group, CCBG, DETEMA, Facultad de Química, Universidad de la República, 11400 Montevideo, Uruguay*

SUPPORTING INFORMATION

Table S.1. CASPT2 equilibrium geometry for thiirane and thioacetaldehyde comparison to CCSD(T), DFT and semi-experimental geometries.[a]

Species	Param.	SE	CASSCF/CASPT2		*ae*-CCSD(T)[a]	ωB97X-D		M06-2X-D3		B2PLYP-D3	
			j-VTZ	VTZ	cc-pwCVQZ	j-VTZ	VTZ	j-VTZ	VTZ	j-VTZ	VTZ
THI [c]	r_{CC}	1.484	1.481	1.482	1.481	1.475	1.477	1.479	1.480	1.478	1.479
	r_{CH}	1.083	1.081	1.081	1.080	1.081	1.081	1.081	1.081	1.080	1.080
	r_{CS}	1.815	1.820	1.816	1.811	1.817	1.811	1.806	1.802	1.827	1.821
	θ_{HCH}	115.8	115.8	115.9	115.1	115.1	115.1	115.3	115.3	115.3	115.3
	θ_{CSC}	48.27	48.0	48.2	48.3	47.9	48.1	48.3	48.5	47.7	47.9
	θ_{CCS}	65.87	66.0	65.9	65.9	66.0	65.9	65.8	65.7	66.1	66.0
	θ_{HCC}	117.9	117.9	117.9	118	118.2	118.1	118	118	118.2	118.1
THA [d]	r_{CS}	1.610	1.627	1.624	1.614	1.612	1.608	1.609	1.606	1.622	1.618
	r_{CH}	1.089	1.087	1.087	1.086	1.088	1.089	1.089	1.089	1.088	1.088
	r_{CC}	1.506	1.500	1.501	1.492	1.496	1.496	1.498	1.498	1.499	1.499
	θ_{CCS}	125.3	124.7	124.7	125.7	125.4	125.5	125.1	125.1	125.3	125.4
	θ_{HCC}	119.4	116.4	116.6	119.7	115.9	115.8	116.0	116.0	116.0	115.9
MARE [e]			0.50%	0.41%	0.29%	0.55%	0.50%	0.44%	0.52%	0.64%	0.55%
MAE len. [f]			0.006	0.004	0.005	0.004	0.004	0.004	0.005	0.007	0.005
MAE ang. [g]			0.67	0.60	0.26	0.85	0.82	0.72	0.77	0.83	0.80

[a] Bond lengths and angles in Å and °, respectively. j-VTZ and VTZ stand for jun-cc-pV(T+d)Z and cc-pVTZ basis sets, respectively
[b] All electrons CCSD(T)/ cc-pwCVQZ from ref. [27] for THI and THA.
[c] Semi-experimental equilibrium geometry from Ref. [91]
[d] Experimental structure from Ref. [93]
[e] Mean Absolute Relative Error over all the structural parameters.
[f] Mean Absolute Error over all bond lengths.
[g] Mean Absolute Error over all bond angles.

Table S.2. Cartesian coordinates and zero-point vibrational energies (ZPVE / Ha) of equilibrium geometries at B2PLYP-D3/jun-cc-pV(T+*d*)Z level of theory for the species on the TSM potential energy surfaces. jun-cheap electronic energies (Eel / Ha) are also reported. All transitions states (TS) present one imaginary frequency (ω_i), the remaining species correspond to minima on the PES with all real frequencies.

**TSM** (Eel = -476.63325, ZPVE = 0.05062)			
C	1.376142	0.448134	-0.000000
S	0.000000	-0.424318	-0.000000
C	-1.376142	0.448134	0.000000
H	1.397314	1.525568	-0.000000
H	2.282715	-0.130927	-0.000000
H	-2.282715	-0.130927	0.000000
H	-1.397314	1.525568	0.000000
**THI** (Eel = -476.69259, ZPVE = 0.05532)			
S	-0.795802	0.000000	-0.000000
C	0.868477	-0.739630	-0.000000
C	0.868477	0.739630	0.000000
H	1.141071	-1.248913	-0.912394
H	1.141071	-1.248913	0.912394
H	1.141071	1.248913	-0.912394
H	1.141071	1.248913	0.912394
**THA** (Eel = -476.69042, ZPVE = 0.05347)			
C	1.733008	-0.252627	0.000000
C	0.453876	0.529090	0.000000
S	-1.032240	-0.110336	0.000000
H	0.557332	1.612532	0.000000
H	2.604464	0.398140	0.000000
H	1.772982	-0.901094	0.874835
H	1.772982	-0.901094	-0.874835
**VTH syn** (Eel = -476.68868, ZPVE = 0.05144)			
C	-0.642740	-0.481243	-0.000000
C	-1.739848	0.272968	-0.000000
S	1.031073	0.047724	0.000000
H	0.787850	1.364220	0.000000
H	-0.715451	-1.560765	-0.000000
H	-2.714759	-0.190270	-0.000000
H	-1.698170	1.352727	0.000000
**VTH anti** (Eel = -476.68818, ZPVE = 0.05111)			
C	0.645282	0.502638	-0.009383
C	1.724497	-0.276117	0.012421
S	-0.999368	-0.129527	-0.008498
H	-1.611786	1.025830	0.264186
H	0.733584	1.577722	-0.067114
H	2.709843	0.161296	-0.035580
H	1.655577	-1.352920	0.071927
**ETH cis** (Eel = -476.61831, ZPVE = 0.04965)			
C	1.610539	0.190623	0.000158
H	1.429771	1.273640	0.000104
C	0.405609	-0.671612	0.000056
S	-1.066905	0.024558	-0.000103
H	2.220174	-0.077721	-0.865616
H	-0.868723	1.393133	-0.000114
H	2.219966	-0.077663	0.866098
**ETH trans** (Eel = -476.61662, ZPVE = 0.05033)			
C	-1.662238	-0.251579	0.000000
H	-1.465770	-1.329217	0.000020
C	-0.496602	0.685329	0.000001
S	0.943252	-0.148893	0.000000
H	-2.279835	0.002978	-0.864517
H	1.806841	0.882090	-0.000003

H	-2.279867	0.003007	0.864486
**MMS cis** (Eel = -476.61423, ZPVE = 0.05078)			
C	1.483459	0.378612	0.000000
S	-0.160503	-0.453078	0.000000
C	-1.415489	0.559100	0.000000
H	-1.114380	1.613230	0.000000
H	1.334582	1.454740	0.000000
H	2.031137	0.070117	0.887799
H	2.031137	0.070117	-0.887799
**MMS trans** (Eel = -476.61853, ZPVE = 0.05146)			
C	-1.154686	-0.855212	0.000000
S	0.001809	0.539169	0.000000
C	1.478075	-0.155876	0.000000
H	2.177062	0.694182	0.000000
H	-0.561844	-1.765324	0.000000
H	-1.780797	-0.796042	0.887034
H	-1.780797	-0.796042	-0.887034
**TS1** (Eel = -476.60522, ZPVE = 0.05047, ω_i = 487i cm^{-1})			
C	-1.165638	-0.629125	0.040471
C	1.165637	-0.629126	-0.040471
S	0.000000	0.607398	0.000000
H	1.875104	-0.769923	0.764020
H	1.072300	-1.373678	-0.812200
H	-1.072301	-1.373678	0.812199
H	-1.875104	-0.769922	-0.764021
**TS2** (Eel = -476.59610, ZPVE = 0.05025, ω_i = 895i cm^{-1})			
C	-1.667528	-0.370283	-0.017277
C	-0.668108	0.607722	0.037101
S	1.043729	-0.111646	-0.024150
H	-0.813353	1.546920	-0.479243
H	-2.506958	-0.315247	-0.698453
H	-1.558041	-1.258268	0.587613
H	-0.422702	0.741276	1.120187
**TS3** (Eel = -476.59614, ZPVE = 0.04730, ω_i = 2059i cm^{-1})			
C	-0.558178	0.625204	0.037070
C	-1.591479	-0.320438	-0.023137
S	0.971642	-0.097889	-0.004530
H	-0.363978	-1.093152	-0.383200
H	-0.688289	1.701778	-0.004005
H	-2.576216	-0.063972	-0.398910
H	-1.600129	-1.068055	0.763914
**TS4** (Eel = -476.56853, ZPVE = 0.04636, ω_i = 1717i cm^{-1})			
C	-1.727573	-0.251110	-0.000000
C	-0.518548	0.612704	-0.000000
H	-2.336035	0.007695	0.869658
H	-1.507775	-1.324323	-0.000000
H	-2.336035	0.007695	-0.869658
S	1.013360	-0.135235	0.000000
H	0.776359	1.293662	-0.000000
**TS5** (Eel = -476.56753, ZPVE = 0.04705, ω_i = 749i cm^{-1})			
C	1.678175	-0.305774	0.022465
C	0.630043	0.707174	0.002149
S	-1.016912	-0.099110	-0.040089
H	-1.060861	-0.000317	1.298832
H	1.444319	-1.297362	0.417870
H	1.750234	-0.417926	-1.082707
H	2.643099	0.080363	0.344689
**TS6** (Eel = -476.58261, ZPVE = 0.04686, ω_i = 1346i cm^{-1})			
C	-1.668919	0.275936	-0.012547
H	-1.605813	1.367654	-0.001366
C	-0.591159	-0.603886	-0.077030

S	0.971602	0.131795	0.004853
H	-1.332690	-0.513379	0.982347
H	1.692476	-0.993818	0.042110
H	-2.666553	-0.136636	-0.110471
**TS7** (Eel = -476.68463, ZPVE = 0.05091, ω_i = 235i cm^{-1})			
C	0.665766	0.505495	0.032889
C	1.720030	-0.301008	-0.018176
S	-1.022519	-0.069563	-0.044284
H	-1.089609	-0.537476	1.207732
H	0.788014	1.578098	0.093288
H	2.721547	0.104000	0.029212
H	1.610981	-1.372588	-0.100575
**TS8** (Eel = -476.59321, ZPVE = 0.04964, ω_i = 238i cm^{-1})			
C	0.708431	0.855086	-0.116608
C	1.071992	-0.523627	-0.022526
S	-0.829092	-0.044764	-0.064769
H	0.887007	1.483413	0.748932
H	-0.943548	-0.666549	1.154300
H	1.203222	-1.111067	-0.919971
H	1.452728	-0.995531	0.888954
**TS9** (Eel = -476.53960, ZPVE = 0.04975, ω_i = 649i cm^{-1})			
C	-1.221448	-0.526418	0.004708
C	-0.423567	1.042596	0.102230
S	0.774550	-0.172786	-0.004667
H	-0.499498	1.535970	-0.877845
H	-0.971650	-1.426217	-0.585287
H	-1.583252	-0.764949	0.990524
H	-1.930371	-0.009404	-0.652622
**TS10** (Eel = -476.55545, ZPVE = 0.04682, ω_i = 1279i cm^{-1})			
C	1.995657	-0.288763	0.000000
C	0.125761	0.841724	0.000000
S	-1.039680	-0.198339	0.000000
H	1.192372	1.149572	0.000000
H	2.951817	0.230861	0.000000
H	1.789539	-0.836181	-0.910570
H	1.789539	-0.836181	0.910570
**TS11** (Eel = -476.54922, ZPVE = 0.04876, ω_i = 408i cm^{-1})			
C	-1.315950	-0.013957	-0.000051
S	0.464983	-0.524603	0.002442
C	0.441273	1.258807	-0.095577
H	0.638724	1.585945	0.945952
H	-1.715021	-0.022974	-1.004891
H	-1.470084	0.980875	0.446125
H	-1.790018	-0.723656	0.673972
**THI triplet** (Eel = -476.52820, ZPVE = 0.04963)			
S	-0.853848	0.000000	-0.000000
C	0.935911	-0.759541	0.000000
C	0.935911	0.759541	0.000000
H	1.199967	-1.279915	-0.912594
H	1.199967	-1.279915	0.912594
H	1.199967	1.279915	-0.912594
H	1.199967	1.279915	0.912594
**I1 singlet** (Eel = -476.59357, ZPVE = 0.05007)			
C	-0.799137	-0.814178	-0.059187
C	-1.052439	0.602625	-0.011307
S	0.777338	0.030299	-0.036065
H	-0.997435	-1.380978	0.844542
H	0.951841	0.649568	1.177749
H	-1.205028	1.151316	-0.930120
H	-1.363110	1.137815	0.891315
**I1 triplet** (Eel = -476.56519, ZPVE = 0.04804)			

C	-1.746763	-0.391722	-0.110548
C	-0.712001	0.607054	0.080644
S	1.005735	-0.090777	-0.045037
H	-2.006708	-1.299079	0.411015
H	0.916181	-0.874576	1.035183
H	-0.745649	1.365671	-0.700776
H	-0.793508	1.123839	1.039385
**I2 triplet** (Eel = -476.60254, ZPVE = 0.05067)			
C	-1.708713	-0.364151	-0.000000
C	-0.632817	0.633151	0.000002
S	1.046913	-0.129837	-0.000001
H	-0.644735	1.280613	-0.877490
H	-0.644727	1.280598	0.877506
H	-2.021228	-0.822595	-0.924689
H	-2.021200	-0.822627	0.924682
**CH2** (Eel = -39.13000, ZPVE = 0.01682)			
H	0.860095	-0.595431	0.000000
C	-0.000000	0.100015	0.000000
H	-0.860095	-0.595431	-0.000000
**H2CS** (Eel = -437.37229, ZPVE = 0.02481)			
H	1.669422	-0.920936	-0.000000
C	1.093971	-0.000000	-0.000000
H	1.669422	0.920936	0.000000
S	-0.515845	0.000000	0.000000
**CH3** (Eel = -39.83183, ZPVE = 0.02998)			
C	0.000000	-0.000004	0.000000
H	-1.072407	0.087374	0.000000
H	0.536204	-0.043675	0.931810
H	0.536204	-0.043675	-0.931810
**HCS** (Eel = -436.71378, ZPVE = 0.01179)			
H	-1.864760	0.675805	0.000000
C	-1.089387	-0.085022	-0.000000
S	0.467658	0.010608	0.000000
**CH4** (Eel = -40.51184, ZPVE = 0.04499)			
H	1.086556	0.000000	0.000000
C	0.000001	0.000000	0.000000
H	-0.362189	0.000000	1.024414
H	-0.362189	0.887168	-0.512207
H	-0.362189	-0.887168	-0.512207
**CS** (Eel = -436.12515, ZPVE = 0.00292)			
C	-1.118615	0.000000	0.000000
S	0.419847	0.000000	0.000000
**CH3CS** (Eel = -476.03592, ZPVE = 0.04151)			
C	-1.796249	-0.156872	0.000000
C	-0.442400	0.421410	0.000000
S	1.044367	-0.072894	0.000000
H	-1.796036	-1.249274	0.000000
H	-2.340045	0.205971	0.873142
H	-2.340045	0.205971	-0.873142
**C2H2** (Eel = -77.32705, ZPVE = 0.02684)			
C	0.601324	0.000000	0.000000
H	1.662117	0.000000	0.000000
C	-0.601324	-0.000000	0.000000
H	-1.662117	-0.000000	0.000000
**H2S** (Eel = -399.30501, ZPVE = 0.01525)			
S	-0.000000	-0.054828	-0.000000
H	0.965918	0.869671	-0.000000
H	-0.965918	0.869671	0.000000
**C2H4** (Eel = -78.58073, ZPVE = 0.05122)			
C	-0.664129	0.000000	0.000000
C	0.664129	-0.000000	-0.000000
H	1.229862	-0.920832	-0.000000

H	1.229862	0.920832	-0.000000
H	-1.229862	-0.920832	0.000000
H	-1.229862	0.920832	0.000000
